# LGR5 promotes invasion and migration by regulating YAP activity in hypopharyngeal squamous cell carcinoma cells under inflammatory condition

**DOI:** 10.1371/journal.pone.0275679

**Published:** 2022-10-26

**Authors:** Zijia Zhu, Shuyuan Yu, Kai Niu, Ping Wang

**Affiliations:** 1 Department of Otolaryngology-Head and Neck Surgery, The First Hospital of Jilin University, Changchun, Jilin, China; 2 Department of Breast Surgery, Jilin University, Changchun, Jilin, China; University of Oklahoma Health Sciences Center, UNITED STATES

## Abstract

High leucine-rich repeat-containing G-protein coupled receptor 5 (LGR5) expression caused by an inflammatory condition was reported to promote tumor proliferation and the epithelial–mesenchymal transition (EMT) in various malignant tumors, but those effects have not been studied in hypopharyngeal squamous cell carcinoma (HSCC) and the molecular mechanism remains unclear. This study was aimed to determine whether YAP/TAZ is involved in the regulation of LGR5 expression in the inflammatory condition. Human hypopharyngeal carcinoma FaDu cells were stimulated with inflammatory medium. The cell invasion ability were evaluated through wound healing assay and transwell invasion assay. The expression levels of EMT-related proteins, LGR5, and p-YAP were detected by real time PCR, western blotting, and immunofluorescence. The results showed that LGR5 expression and the EMT process were significantly enhanced under inflammatory condition. The expression of EMT-related proteins was up-regulated, while that of p-YAP was decreased. After inhibiting the high LGR5 expression with short interfering RNA, the expression of EMT-related proteins was also down-regulated, while that of p-YAP was significantly increased. The use of verteporfin (VP), an inhibitor of YAP activity that promotes YAP phosphorylation, did not affect LGR5 expression. In conclusion, we suggest that the inflammatory condition leads to high LGR5 expression, which up-regulating the expression of EMT-related proteins by inhibiting the YAP phosphorylation.

## Introduction

The metastasis of hypopharyngeal squamous cell carcinoma (HSCC) is an important cause of its poor prognosis. In the past 20 years, the 5-year survival rate of patients with HSCC has never exceeded 55%. Because of the abundant blood supply and lymphatic drainage pathway around the head and neck, malignant tumors in this area are often prone to early metastasis.

In recent years, studies on malignant tumor infiltration, distant metastasis, and the epithelial–mesenchymal transition (EMT) have attracted much attention. Epithelial cells lose their original polarity, express the characteristics of mesenchymal cells, and acquire the ability of migration and anti-apoptosis. EMT is considered an important early event of tumor metastasis and is the main mode of metastasis and invasion of most malignant tumors [1]. Inhibition of the invasion and metastasis of tumor cells can be a new method for the treatment of squamous cell carcinoma. The underlying mechanism of EMT in head and neck tumors remains unclear and is of great research value.

The tumor development process is not only limited to local behavior but interactions with peripheral blood vessels, the extracellular matrix, surrounding normal cells, and related signaling molecules to form a complex regulatory network, namely the tumor microenvironment. Various signals in the tumor microenvironment play important roles in the survival, self-renewal, invasion, and metastasis of tumor stem cells [2]. The major components of the tumor microenvironment include the hypoxia microenvironment and inflammatory condition.

The inflammatory condition and immunosuppressive condition are two important components of the tumor microenvironment. The infiltration of immune cells and aggregation of inflammatory factors can induce the activation of oncogenes, promote the formation of tumor blood vessels, and promote the invasion, development and even metastasis of tumors [3, 4]. Leucine-rich repeat-containing G-protein coupled receptor 5 (LGR5), also known as GPR49, is a member of the G protein-coupled receptor protein family. LGR5 was initially found in the small intestine and hair follicles, is a marker of adult intestinal epithelial stem cells, and plays a crucial role in embryonic development [5]. In recent years, many studies have shown that LGR5 is highly expressed in multiple malignant tumor types, including colorectal cancer, ovarian cancer, hepatocellular carcinoma, basal cell carcinoma, and esophageal adenocarcinoma. Overexpression of LGR5 is significantly correlated with advanced clinical stage and metastatic status of breast cancer, indicating that LGR5 may be a promising prognostic marker for patients with breast cancer [6]. Studies have shown that LGR5 promotes EMT by activating the Wnt/β-catenin pathway, which promotes the invasion and migration of breast cancer, glioma stem cells, and a variety of other malignant tumors [7, 8]. LGR5 is expressed differently in different tissues but has not been studied in hypopharyngeal cancer. The mechanism of action and regulation of different inflammatory factors secreted by inflammatory cells concerning the EMT phenotype of head and neck squamous epithelial cells under continuous inflammatory stimulation, as well as the correlation between the phenotypic transformation of LGR5+ stem cells and acquisition of EMT ability, warrant further study. In this study, the inflammatory cell supernatant produced by THP-1 macrophages under lipopolysaccharide (LPS) stimulation was applied to head and neck tumor cell lines, to establish the inflammatory condition model of tumor cells, to isolate the subpopulation of cells with high LGR5 expression induced by inflammatory factors, and to determine the correlation between LGR5 and EMT by comparing and analyzing the EMT phenotypic transformation ability with parental cells. By constructing a recombinant LGR5 plasmid to transfect tumor cells to overexpress LGR5 protein, the regulatory pathways and molecular mechanisms of EMT and phenotypic transformation of stem cells induced by inflammatory factors were verified.

YAP/TAZ is an important pair of transcription regulators that are highly active in most human malignancies. Recent studies have shown that YAP/TAZ plays a very important role in tumor development [9]. As an upstream or downstream factor of multiple signaling pathways, YAP/TAZ ultimately promotes tumor proliferation, invasion, and angiogenesis and metastasis. Normally, in mammals, the Hippo pathway kinase cascade leads to the phosphorylation of YAP/TAZ, trapping them in the cytoplasm, blocking their interaction with the TEA domain family member 1 (TEAD) family of transcription factors, and inhibiting cell proliferation and malignant transformation [10]. However, non-phosphorylated YAP/TAZ tends to be increased significantly in malignant tumor cells; they can enter the nucleus smoothly and regulate the expression of several genes related to proliferation, anti-apoptosis, and stem cell characteristics [11], including the secretory proteins connective tissue growth factor (CTGF) and CYR61 [12, 13], AXL receptor tyrosine kinase, c-myc and surviving [14]. As a type of malignant tumor that seriously endangers human health, the role of YAP/TAZ in head and neck tumors has been rarely studied. Since both LGR5 and YAP/TAZ are closely related to tumor development, invasion and metastasis ability as well as stem cell characteristics, and researchers have found their regulatory relationship in studies of colon cancer, we believe that there may be also a certain relationship between the two in hypopharyngeal squamous cell carcinoma. Therefore, verteporfin (VP), a benzoporphyrin derivative belonging to the porphyrin family, is a YAP inhibitor used in this study to disrupt the YAP-TEAD interaction [15].

It is important to clarify the complex relationship between LGR5/YAP signaling and the development of hypopharyngeal squamous cell carcinoma. Therefore, we investigated the expression change of LGR5 in FaDu cells under the inflammatory condition. The invasion ability of FaDu cells was evaluated with LGR5 over-expression or knock-down treatment. This study tried to clarify the mechanism of LGR5/YAP signaling regulation in HSCC proliferation and invasion. The data could provide a special target for the clinical treatment of head and neck tumors.

## Methods

### Cell culture

The human hypopharyngeal squamous cancer cell line FaDu and monocyte leukemia cell line THP-1 were obtained from Shanghai Zhongqiaoxinzhou Biotech Co., Ltd. (Shanghai, China). All the cells were grown in RPMI 1640 medium supplemented with 10% fetal bovine serum (FBS; Hyclone Laboratories, Inc., Logan, UT, USA), 1% penicillin, and 1% streptomycin and were maintained at 37°C in a humidified incubator with 5% CO2.

### Preparation of FaDu cells with LGR5 over-expression

FaDu cells were transfected with the LGR5 plasmid or control plasmid, which was purchased from GeneCopoeia (EX-Q0041-M03, EX-NEG-M03; Guangzhou, China). The LGR5 recombinant plasmid was constructed using pReceiver-M03 as the backbone plasmid and was inserted into the LGR5 cDNA coding sequence (NM_003667, ORF length: 2,724 bp). FaDu cells transfected with the LGR5 plasmid were cultured in medium containing G418 for 14 d. The LGR5+ FaDu cells were collected to identify LGR5 over-expression using quantitative polymerase chain reaction (qPCR) and western blot assay.

### Short interfering RNA (siRNA) transfection

FaDu cells were stimulated with inflammatory factors and then were transfected with siRNA oligo targeting the LGR5 gene, with a scrambled sequence as the control. Eight hours after transfection, the cell culture medium was replaced with RPMI 1640 containing 10% FBS and the cells were continued to incubate at 37°C for another 40 hours before the next step. A siRNA interference oligo sequence targeting the human LGR5 gene sequence (LGR5-homo-883) was designed and synthesized by Shanghai GenePharma Co., Ltd. The target sequence was UAAUAAGAG AAG GGUUGCCTT.

### Cell proliferation assay

The proliferation ability of cells was measured by the CCK-8 assay using Cell Counting Kit (Yeasen, Shanghai). Different groups of cells were inoculated into 96-well plates at the density of 1,000 cells per well. After 24 hours, the CCK-8 reagent was added, followed by incubation at 37°C for 1 hour. The absorbance at 450 nm was recorded using a microplate reader.

### Immunofluorescence staining

The cells growing on glass coverslips Cells to be observed were inoculated on glass slides, were fixed in 4% paraformaldehyde at room temperature for 30 minutes. The cells were washed three times with PBS and then were incubated with 0.1% Triton X-100, blocked in 5% goat serum for 1 hour, and then were incubated overnight with 1:100 diluted primary antibody at 4°C. Next, the cells were washed with PBS and then were incubated with 1:400 diluted secondary antibody in the dark for 1 hour at room temperature, followed by washing three times with PBS. The slides were sealed with glycerine at a concentration of 50% and then were observed and photographed with a laser confocal microscope (Fluo-View FV1000; Olympus, Japan). The following product was used in this experiment: ①Goat anti-Mouse IgG (H+L) Highly Cross-Adsorbed Secondary Antibody, Alexa Fluor 488 from Thermo Fisher Scientific, catalog # A-11029. ②Goat anti-Rabbit IgG (H+L) Cross-Adsorbed Secondary Antibody, Alexa Fluor 555 from Thermo Fisher Scientific, catalog # A-21428.

### Wound healing assay

FaDu cells of different groups were inoculated in 24-well plates and scratched with a 200-μl pipette tip when the plate was completely covered. PBS solution was used to wash away floating cell debris. Next, photos of the freshly scratched surfaces were taken. Thereafter, the cells were cultured at 37°C and 5% CO2 for 24 hours and then were photographed again to compare the healing of each “wound”. The scratch spacing at each time point was measured by Image J software [RM = (W2-W1)/W2×100% (RM = relative mobility, W1 = initial cell covering rate, W2 = final cell covering rate)]. The relative mobility of the cells at each time point was calculated with 0-hour scratch spacing as a reference, and the experimental results were analyzed using SPSS17.0 statistical software. The counting data were expressed as a percentage, while the measurement data were expressed as ±S. One-way analysis of variance was used for comparison of multi-group mean values.

### Cell migration assay

Cell invasion ability was determined by the Transwell assay. Cell suspensions with concentrations of 5×10^5^/ml were prepared with 0.1% FBS in RPMI 1640 medium. Next, 5×10^4^ cells were placed in the upper chamber (pore size: 8 μm; Millipore, Billerica, MA, USA) precoated with 1:4 diluted Matrigel (Yeasen, Shanghai, China). The lower chamber was filled with RPMI 1640 medium containing 20% FBS as an inducer. After incubation for 24 hours, the cells in the upper chamber and Matrigel were erased with cotton swabs, and the lower cells were stained with 0.5% crystal violet. Cell invasion was observed under an optical microscope (magnification, ×40).

### Real-time quantitative PCR (qPCR)

Total RNA was extracted from each group using Trizol reagent, and the RNA integrity was verified by agarose gel electrophoresis. The RNA was reversed transcribed into cDNA using the Transcriptor First Strand cDNA Synthesis Kit (Cat.04897030001). Additionally, cDNA was amplified using FastStart Essential DNA Green Master, Cat.06924204001, (Roche, Indianapolis, IN, USA). The specific primer designs are shown in S1 Table. The real-time PCR protocol was performed according to our previous studies [16]. The target gene mRNA level was normalized to that in β-actin, and the relative mRNA levels were calculated using the formula: Fold change = 2^−△△CT^.

### Western blot analysis

The proteins were extracted with cell lysate buffer (T-PER Tissue Protein Extraction Reagent containing phosphatase inhibitor (Halt Protease and Single-Use Inhibitor Cocktail; 78442; Thermo). The proteins were separated using 10% SDS-polyacrylamide gel electrophoresis and then were transferred to PVDF membranes. The membrane was blocked with 5% non-fat milk powder for 1 hour and then was incubated with antibodies overnight at 4°C, washed three times with TBST, and then incubated with horseradish peroxidase-labeled secondary antibody at room temperature for 1.5 hours. The primary antibodies were as follows: LGR5 Polyclonal antibody (Cat# A10545), NCD(Cat# A19083), YAP Polyclonal antibody (Cat# A1001), Phospho-YAP1-S127 Polyclonal antibody (Cat# AP0489), and SNAIL Polyclonal antibody (Cat# A5243), all from ABclonal Biotechnology Co., Ltd, China. GAPDH monoclonal antibody (Cat# ab8245; Cambridge, MA, USA) was used as a working internal control. ECL reagent (Thermo Fisher Scientific, Waltham, MA, USA) was used to visualize protein bands, and images were obtained using a Dolphin-C hemi Mini image system (Wealtec Corp., Sparks, NV, USA). Gray values for each protein band were analyzed using Image J analysis software, and relative protein levels were calculated using GAPDH as an internal control.

### Statistical analysis

All the data were collected from at least three independent experiments. Results were analyzed using GraphPad Prism 8.0 statistical software and are expressed as means ± standard deviation (SD). Student’s t-test, one-way analysis of variance, and multivariate analysis of variance were used to analyze data as appropriate. A value of P < 0.05 was considered statistically significant.

## Results

### The inflammatory condition affects the proliferation and migration ability of FaDu cells

**Fig 1:**
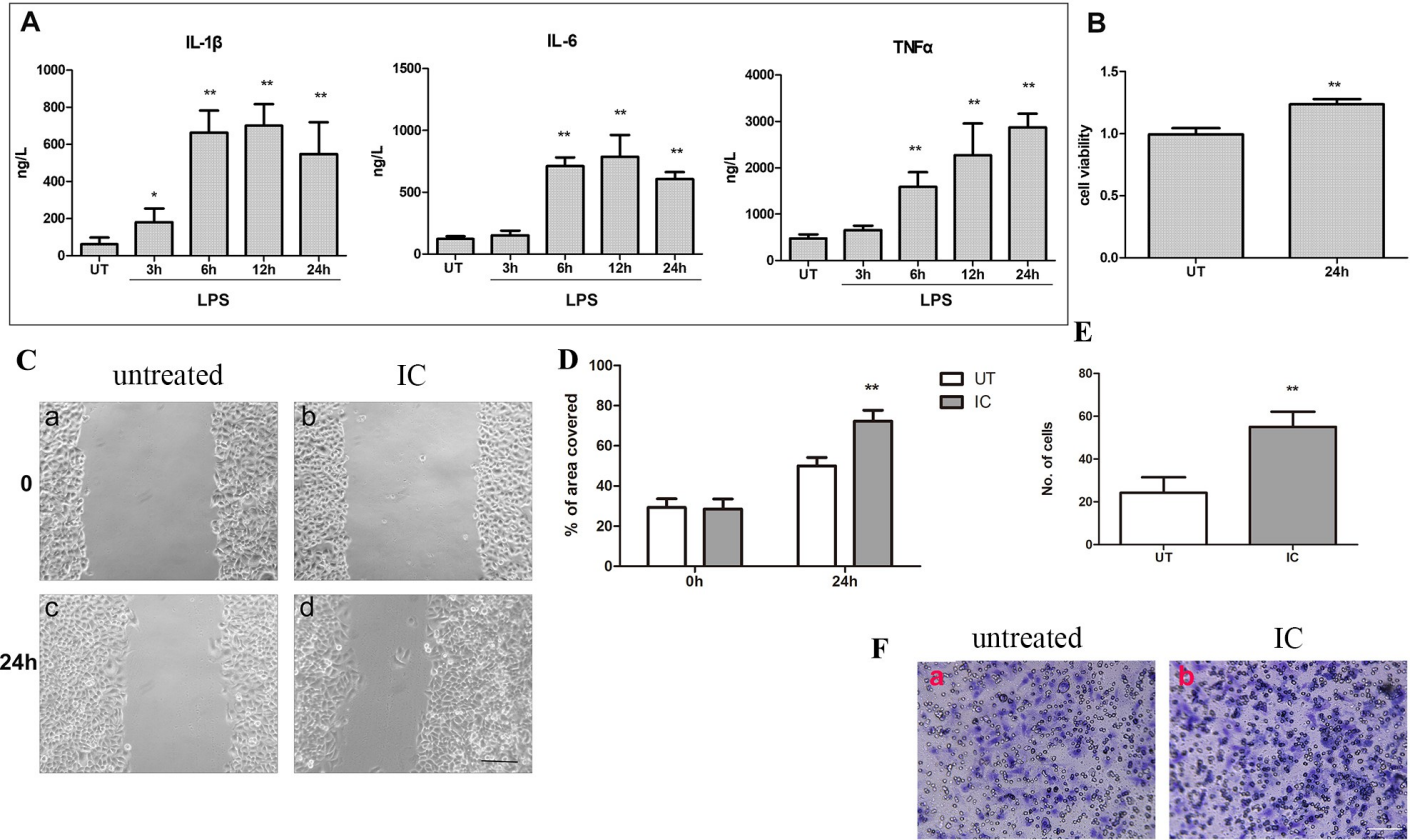
Effect of the inflammatory condition on tumor cell invasion and proliferation. A: Change in the IL-1β, IL-6, and TNF-α levels in THP-1 cells under LPS stimulation. B: Cell viability under the inflammatory condition after 24 hours using the CCK8 assay. C-D: Cell migration was observed under the inflammatory condition after 24 hours using the wound healing assay. Scale bar = 50 μm. E-F: Cell invasion was observed using the Transwell chamber set. Scale bar = 20 μm. Statistical significance is denoted by *P<0.05, **P<0.01, IC group vs UT group. UT: untreated group; IC: inflammatory condition.

By adding LPS (2μg/ml) to the growth media of THP-1 cells, we were able to produce culture medium that was high in cytokines (Fig 1A). We chose to harvest the supernatant at 24 hours after stimulation because the amount of tumor necrosis factor (TNF)-α, the most abundant cytokine, reached its peak at this time. We tried to dilute the supernatant in various proportions, but the CCK-8 assay showed that the diluted supernatant had little effect on tumor cell viability. The medium was then used to simulate an inflammatory condition. The cell viability of FaDu cells treated with the inflammatory condition was compared with that of the untreated control group by the CCK-8 assay. The optical density (OD) of each group was measured 24 hours after administration at a 450-nm wavelength. The OD value was significantly increased in the treatment group. The proliferation of FaDu cells in the experimental group was 24.36% higher than that in the control group. Hence, the results showed that the inflammatory condition can promote the proliferation of FaDu cells (Fig 1B). We also conducted wound healing experiments, which showed that the proliferation and migration of tumor cells in the inflammatory condition were significantly stronger than those of the control group. At 24 hours, the relative mobility of cells in the blank control group was 50.00±4.243%, while that in the experimental group was 72.25±5.439%. A significant difference was found between the groups (P<0.01) (Fig 1C and 1D). Finally, the Transwell invasion assay was performed. This experiment indicated that the ability of HSCC to invade under the stimulation of the inflammatory condition was significantly enhanced compared with that in the control group. The number of transmembrane cells in the experimental group was (55.00±7.071)/HP while that in the control group was (24.20±7.294)/HP (Fig 1E and 1F).

### The inflammatory condition leads to high LGR5 expression in FaDu cells

**Fig 2:**
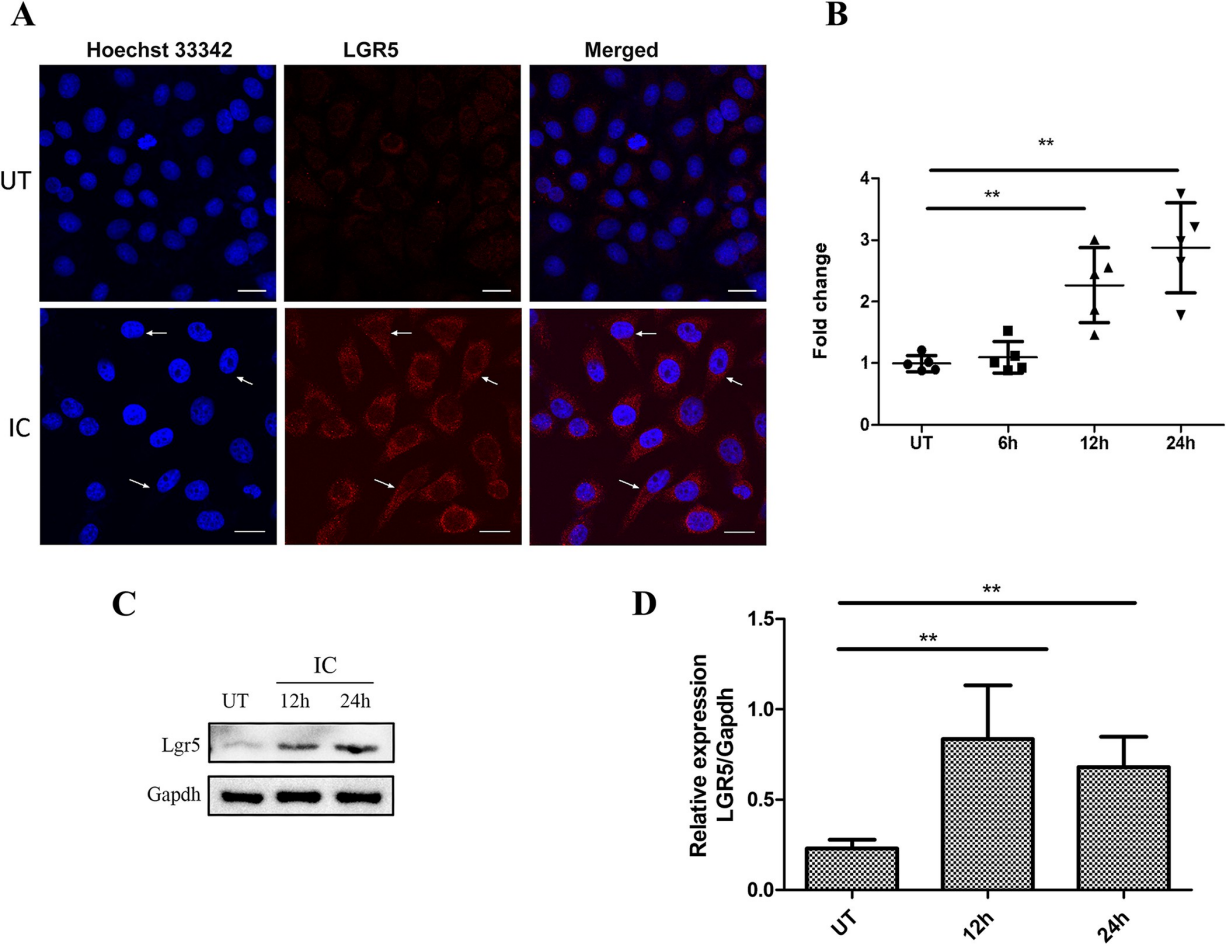
The inflammatory condition affects LGR5 expression in FaDu cells. A: Representative imaging of LGR5 expression in FaDu cells by immunofluorescence assay. FaDu cells stained for Hoechst 33342 (blue) and LGR5 (red) after treatment with the inflammatory condition or control group are shown. The arrows indicate FaDu cells with stromal-like phenotypic changes. Scale bar = 20 μm. B: The expression level of LGR5 in FaDu cells was analyzed by reverse transcription-quantitative PCR after 6, 12, and 24 hours of treatment with the inflammatory condition. C-D: The experimental group was treated with the inflammatory condition for 12 and 24 hours. Western blotting was used to analyze LGR5 expression in FaDu cells. Quantification (D) and representative blots (C) are shown. Statistical significance is denoted by **P<0.01. UT: untreated group; IC: inflammatory condition.

The experimental results of immunofluorescence showed that, compared with the control group, LGR5 expression in the treatment group was significantly higher. Additionally, the morphology of tumor cells in the inflammatory condition showed clear transformation. The cell morphology changed from oval to spindle shaped, giving them a stronger ability to invade (Fig 2A). qPCR was used to detect LGR5 expression. The experimental results showed great changes after stimulation of the inflammatory condition. The LGR5 expression level increased significantly compared with that of the original value (Fig 2B). Using actin as the internal control, the LGR5 mRNA expression level in the experimental group was increased by 2.27 times for 12 hours and 2.88 times for 24 hours compared with the initial value (P<0.01; Fig 2B). Additionally, we conducted western blotting experiments to detect LGR5 expression. The results showed that its expression level was upregulated in the inflammatory condition (Fig 2C and 2D). Compared with the control group, the expression level of LGR5 protein in the experimental group was increased by 3.64 times for 12 hours and by 2.97 times for 24 hours (P<0.01).

### The expression levels of EMT-related genes in FaDu cells with the over-expression or gene-knockdown of LGR5

**Fig 3:**
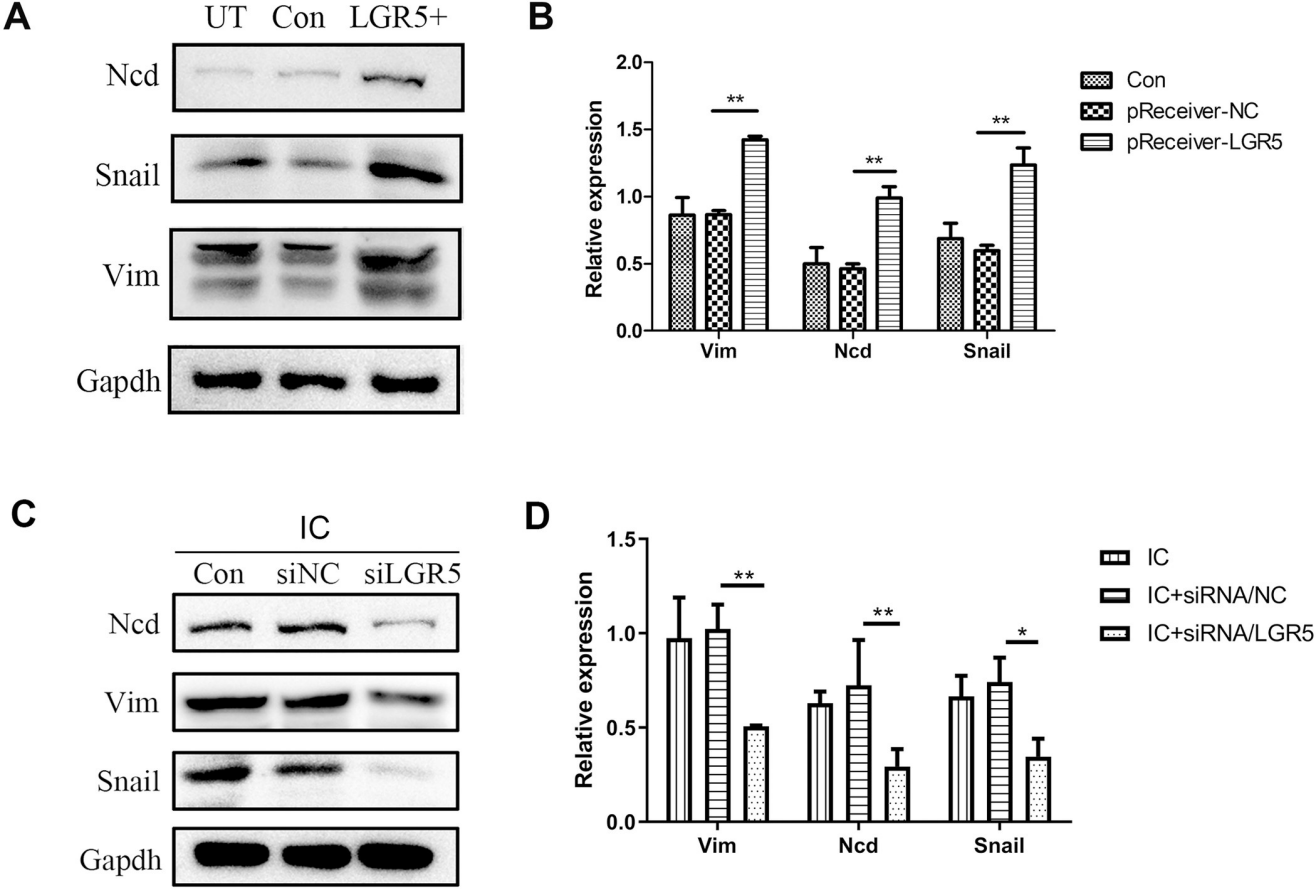
Expression levels of EMT-related genes. A-B: Protein levels of EMT-related genes vimentin, NCD, and snail were determined by immunoblotting illustrating higher protein levels of them in FaDu/LGR5+ cells. GAPDH served as loading control. Quantification (B) and representative blots (A) are shown. C-D: FaDu cells with IC stimulation were transfected with LGR5 siRNA or siRNA NC. Western blotting was used to examine the levels of vimentin, NCD, and snail expression. When LGR5 was silenced with siRNA, the expression of vimentin, NCD, and snail was also decreased. Quantification (D) and representative blots (C) are shown. Statistical significance is denoted by *P<0.05, **P<0.01. UT: untreated group; IC: inflammatory condition; siNC: negative control siRNA; siLGR5: LGR5 siRNA.

To investigate the biological significance of LGR5 in HSCC, the cells were transfected with LGR5 overexpression plasmids. The relative expression levels of EMT-related proteins were measured by western blotting. The levels of vimentin, N-cadherin(NCD), and Snail in the LGR5 overexpression group were markedly upregulated compared with those in the control group. The expression levels of Vimentin, NCD, and Snail were increased by 1.65, 1.98, and 1.80 times, respectively (Fig 3A and 3B). The expression levels of vimentin, NCD, and snail in FaDu cells transfected with LGR5 siRNA or siRNA NC were also detected by western blotting. After IC stimulation of Fadu cells, LGR5 expression was repressed with siRNA, and it was discovered that vimentin, NCD, and snail levels were reduced by 47%, 52%, and 52%, respectively, in the LGR5 siRNA group compared to the siRNA NC group (Fig 3C and 3D).

### Over-expression or gene-knockdown of LGR5 influenced the ability of migration and invasion of FaDu cells in vitro

**Fig 4:**
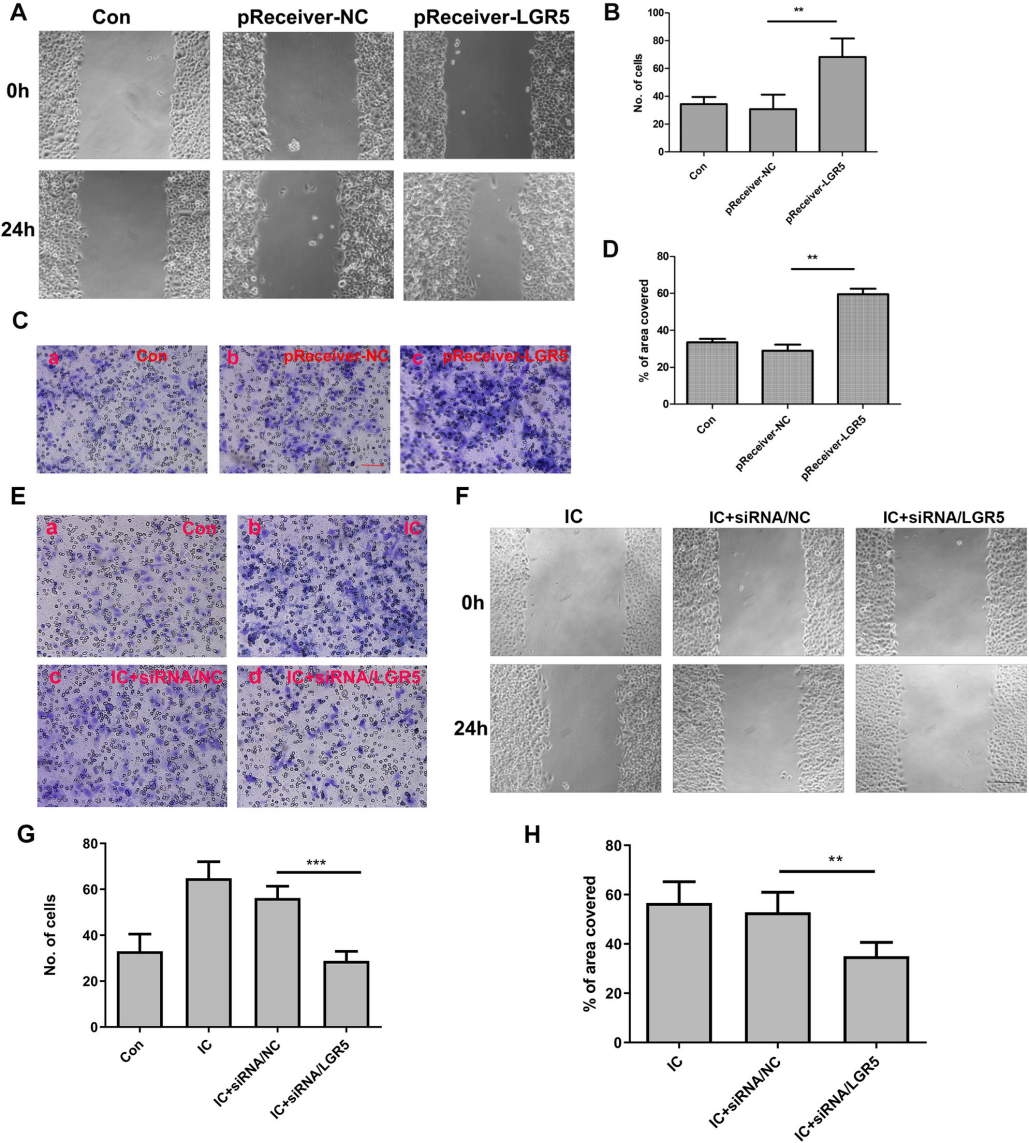
Effects of LGR5 overexpression on tumor cell invasion and proliferation. A, D, F, H: The effect of LGR5 expression on cell migration was detected by the wound healing assay. The percentage of wound healing distance between cell scratches in each group was compared after 24 hours. The outcomes demonstrated that following LGR5 overexpression, the capacity for cell migration was improved. Quantification (D) and representative imaging (A) are shown. Scale bar = 50 μm. B, C, E, G: The Transwell assay was used to test the invasiveness of the cells. According to the quantitative (B) and representative imaging(C) results, both LGR5 overexpression and the inflammatory condition can lead to enhanced cell invasion ability. However, when LGR5 expression is inhibited, the invasion ability stimulated by IC was reduced. Scale bar = 20 μm. Statistical significance is denoted by **P<0.01,***P<0.001. Con: control group; pReceiver-NC: negative control plasmid; pReceiver-LGR5: LGR5 overexpression plasmid; IC: inflammatory condition; siRNA/NC: negative control siRNA; siRNA/LGR5: LGR5 siRNA.

The invasion and migration of HSCC cells were significantly enhanced after they were transfected with the LGR5 overexpression plasmid. The relative mobility of the cells and number of transmembrane cells in the blank control group were 33.40±4.506% and (34.40±5.177)/HP, respectively, while the values in the experimental group transfected with the LGR5 overexpression plasmid were 59.60±6.542% and (68.20±13.41)/HP, respectively. In the group of cells transfected with empty plasmids, the two values were 29.00±7.176% and (30.80±10.40)/HP, respectively (P<0.01) (Fig 4A–4D). As shown in Fig 4F and 4H, the relative mobility of cells in the empty plasmid-transfected group was 52.80±8.167% while that of cells stimulated only by the inflammatory condition was 56.60±8.620%. Additionally, little difference was found between the groups. Nevertheless, when the expression of LGR5 was inhibited, the inflammatory condition cannot enhance cell migration and invasion. As shown in Fig 4E–4H, the relative mobility of cells and number of transmembrane cells were 35.00±5.612% and (28.80±4.147)/HP, respectively. However, the number of transmembrane cells that were stimulated by the inflammatory condition without LGR5 inhibition was (64.80±7.225)/HP (P<0.001).

### LGR5 inhibits the phosphorylation of YAP

**Fig 5:**
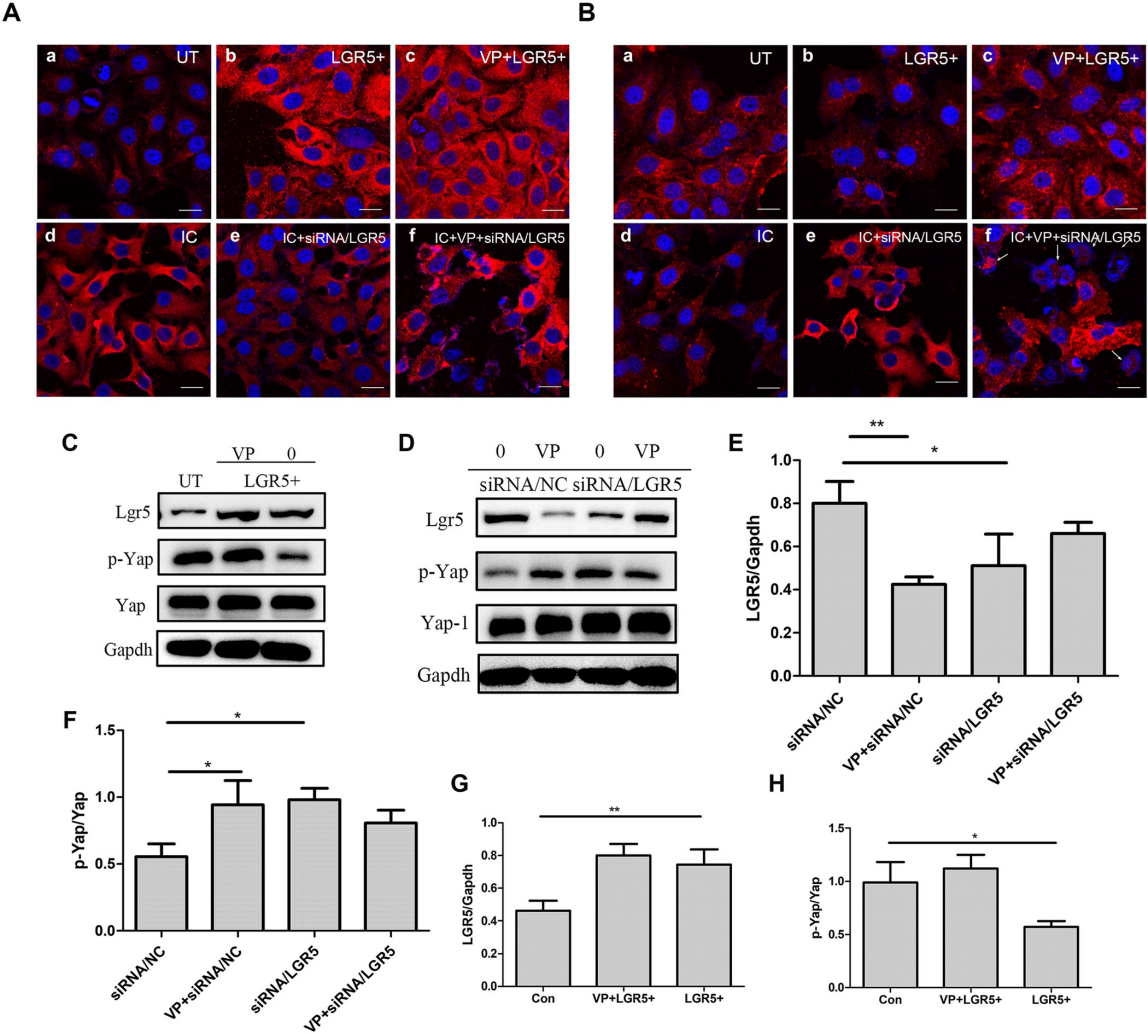
Relationship between the phosphorylation of YAP and LGR5 expression. A: LGR5 expression was detected by immunofluorescence assay. FaDu cells were subjected to immunofluorescent staining for Hoechst 33342 (blue) and LGR5 (red). The cells were subjected to various treatments: transfection with LGR5 plasmids or stimulation with the inflammatory condition; inhibition of LGR5 expression by siRNA; inhibition of YAP phosphorylation by VP. The experimental results of immunofluorescence of LGR5 expression are shown in A,Scale bar = 20 μm. The expression of p-YAP was detected by immunofluorescence assay. The results of p-Yap expression are shown in B. FaDu cells were subjected to immunofluorescent staining for Hoechst 33342 (blue) and phosphorylated YAP (red). C-H: Western blotting was used to detect LGR5 or p-YAP expression in LGR5-overexpressed cells or siRNA/LGR5-transfected FaDu cells. Statistical significance is denoted by *P<0.05 **P<0.01. UT: untreated group; IC: inflammatory condition; VP: verteporfin; LGR5+: stable cell lines overexpressing LGR5; siRNA/NC: negative control siRNA; siRNA/LGR5: LGR5 siRNA.

The expression of LGR5 and p-YAP was detected by immunofluorescence and western blotting. The phosphorylation level of YAP was decreased by 58% when LGR5 was overexpressed compared with that in the control group (P<0.001) (Fig 5B, 5C and 5H). However, after the addition of siRNA to inhibit the high expression of LGR5 caused by the inflammatory condition, the phosphorylation level of YAP was 1.76 times higher than that of cells treated with the inflammatory condition alone (P<0.001) (Fig 5A, 5D–5F). The use of VP to increase the phosphorylation level of YAP did not affect LGR5 expression (Fig 5F and 5H).

Regarding immunofluorescence analysis, after inflammatory condition stimulation, siRNA transfection, and VP treatment, many cells died, and obvious nuclear fragmentation was observed (Fig 5A–5f, 5B–5f). We speculate that siRNA may be the cause of cell death since it has a potent inhibitory effect on LGR5 expression. Low LGR5 expression results from this, which lowers the tolerance of cells to VP and ultimately causes cell death.

### VP impairs the ability of FaDu cells to invade and migrate

**Fig 6:**
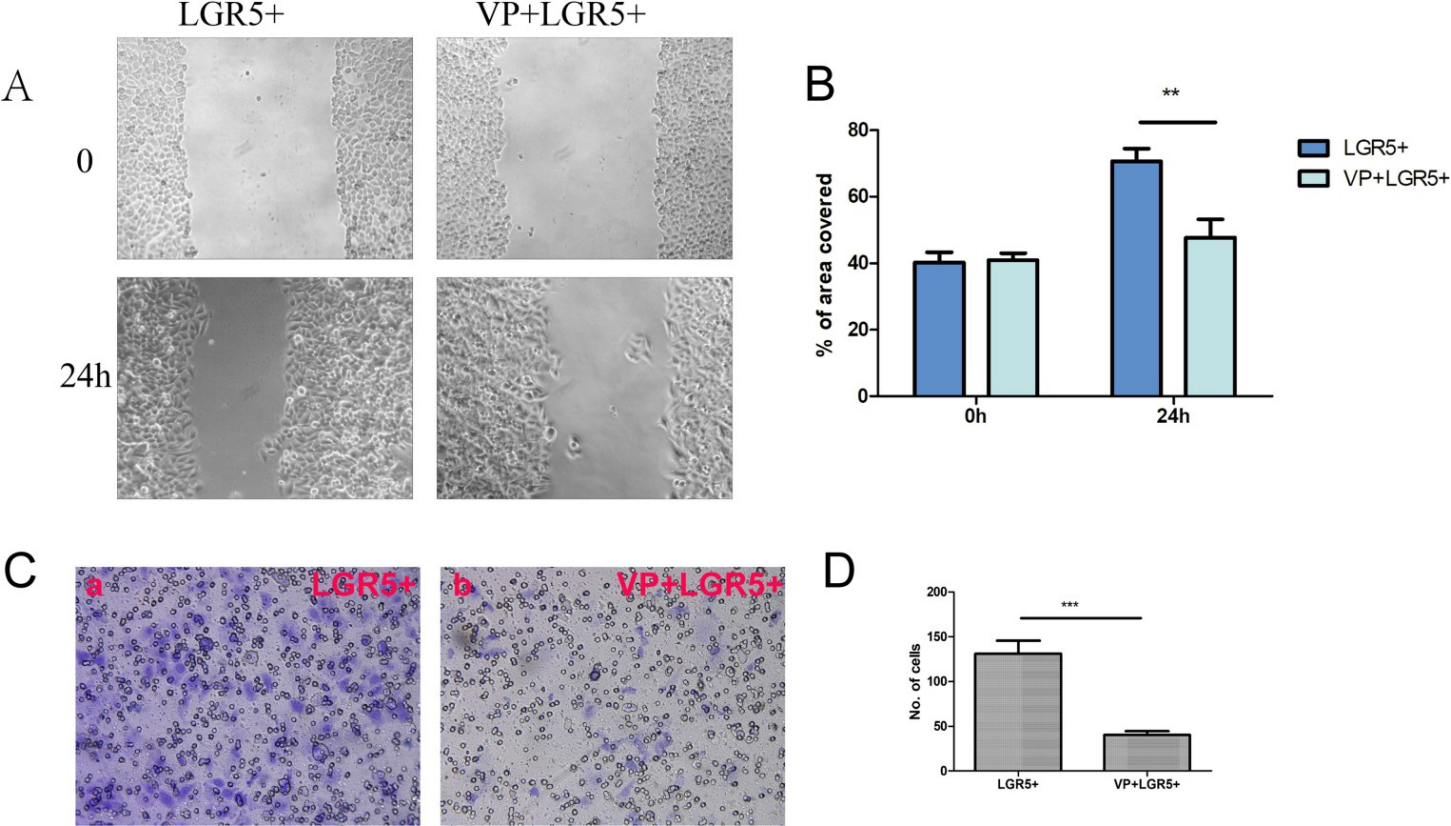
VP affects the invasion and migration of FaDu cells. A-B: The ability of cell migration was measured by the wound healing test. Quantification (B) and representative imaging (A) are shown. Scale bar = 50 μm. C-D. The invasiveness of FaDu cells was detected by the transwell assay. The pore diameter of the chamber is 8 μm. Quantification (D) and representative imaging (C) are shown. After 24 hours, each experimental group was compared in terms of the percentage of wound healing distance between cell scratches or the number of migrating cells. Scale bar = 20 μm. The mean between the groups was compared by independent samples t-test. Statistical significance is denoted by **P<0.01,***P<0.001.

We conducted wound healing experiments, and the results showed that the migration of LGR5-overexpressed FaDu cells after VP stimulation was weaker than that of the control group. At 24 hours, the relative mobility of cells in the control group was 131.0±14.53% while that in the VP experimental group was 40.33±4.163% (P<0.01; Fig 6A and 6B). Next, the Transwell invasion assay was performed. The invasive ability of LGR5-overexpressed FaDu cells after VP stimulation was predominantly diminished compared with that in the control group. The number of transmembrane cells in the experimental group with VP was (47.67±5.508)/HP while that in the control group was (70.67±3.786)/HP (Fig 6C and 6D).

## Discussion

High LGR5 expression has been previously reported to be closely related to tumor proliferation and invasion and has been detected in various malignancies. Studies on liver cancer, colorectal tumors, basal cell carcinoma, ovarian cancer, and other tumors [17] have shown that LGR5 promotes cell proliferation and invasion in tumor cells, and leads to tumor stem cell-like characteristics, resulting in a poor prognosis. Other studies have shown that LGR5 plays an inhibitory role in colorectal cancer (CRC) progression in colorectal cancer [18]. In terms of oral squamous cell carcinoma (OSCC) in head and neck tumors. Some researchers suggest that LGR5 overexpression may contribute to clonal expansion during OSCC development through LGR5-mediated increased Wnt signaling. And the high expression of LGR5 was to be considered as an independent prognosis factor for patients’ overall survival time [19, 20]. LGR5 expression in different tumors is different while that in hypopharyngeal cancer has not been studied. A large number of researchers have also focused heavily on the link between inflammation and the occurrence and growth of malignant tumors. For instance, Khalili M. et al. demonstrated that the prostatic inflammatory milieu drastically reduced the expression level of the important tumor suppressor gene Nkx3.1, demonstrating that inflammation was intimately linked to oxidative stress and carcinogenesis [21]. Similar investigations into the connection between the inflammatory conditions and malignent tumors in the liver [22] and colon [23] have been made.

Inflammatory condition contains numerous inflammatory agents, including interleukin (IL)-1, IL-6, and TNF-α. Studies have shown that these cytokines are important factors that promote the generation, proliferation, and invasion of various tumor cells [24, 25]. In studies related to IL-1, B16 melanoma cells were inoculated with wild-type and IL-1β-knockout (KO) mice respectively. The IL-1β-knockout mice did not develop tumors, but the wild-type mice developed melanoma, which caused deaths [25]. Thus, the inflammatory condition plays a key role in the occurrence and development of malignant tumors. In this study, LPS was used to stimulate THP-1 cells to produce inflammatory factors to simulate the inflammatory condition. The proliferation and invasion of FaDu cells were enhanced in the inflammatory condition. After the inflammatory condition was used to stimulate FaDu cells, LGR5 expression was detected by western blotting. After 12 hours of stimulation, LGR5 expression was significantly up-regulated and did not continue to increase over time (Fig 2C and 2D). However, when siRNA was used to inhibit the high LGR5 expression in FaDu cells caused by inflammatory conditional stimulation, the cell viability was significantly reduced, and the ability of proliferation and invasion was weakened. Therefore, the inflammatory condition likely causes high LGR5 expression in HSCC, thus promoting the proliferation and invasion of tumor cells.

We confirmed through scratch and Transwell experiment that high LGR5 expression can promote the migration and invasion of tumor cells. Subsequently, we detected the expression of various EMT-related proteins, among which the expression levels of vimentin, NCD, and snail were regulated by LGR5. In several cancers, including stomach, colon, and breast tumors, ECD is crucial for EMT [26, 27]. The expression of ECD in the inflammatory condition of Fadu does not, however, alter significantly due to tissue specificity.

Previous studies have shown that the YAP-TEAD interaction is an important factor that promotes the proliferation, migration, and invasion of tumor cells [28, 29]. Many studies have demonstrated that the hyperfunction of YAP and TAZ may promote the proliferation of tumor cells [30]. and the process of EMT, which includes high expression of YAP/TAZ or nuclear enrichment, has been detected in melanoma, breast cancer, and other tumors [31]. YAP can be transported to the nucleus and bind with transcription factors such as TEADs to promote the expression of target genes [32]. The YAP/TAZ pathway is associated with the expression of many genes. For example, studies have shown that VASN (vasorin) in thyroid cancer promotes the EMT of thyroid cancer cells by triggering the YAP/TAZ pathway [33]. TRPV4 (transient receptor potential cation channel subfamily V member 4) was found to be very important for the nuclear transport of YAP/TAZ. By regulating YAP/TAZ nuclear translocation, TRPV4 promotes EMT in normal mouse primary epidermal keratinocytes, resulting in changes in the expression levels of EMT-related markers, such as E-cadherin, N-cadherin, and α-smooth muscle actin [34, 35]. In the study of gastric cancer cells, knockout of WBP2 increased p-YAP, which resulted in the retention of YAP in the cytoplasm, inhibited the cross-linking process of YAP-Tead, and finally down-regulated the expression of TEAD target genes CTGF and CYR61 [36]. To sum up, YAP is regulated by the expression products of some genes and can regulate the expression of others. Therefore we considered whether a relationship exists between LGR5 and YAP-TEAD in HSCC just like it is in colorectal cancer [37]. Additionally, studies have shown that specific siRNA silencing of YAP in the human colon cancer cell line HCT116 can affect the recruitment of p300 protein, reduce the acetylation of p53AIP1 target genomic protein, and lead to the delay or reduction of p73-mediated apoptosis [38]. Thus, YAP has different effects in different tumors. The tumor we studied was hypopharyngeal squamous carcinoma. Using various experiments, we proved that the inflammatory condition-induced high LGR5 expression in FaDu cells and LGR5 overexpression lead to the hyperfunction of YAP/TAZ and enhanced proliferation, migration, and invasion of tumor cells.

**Fig 7:**
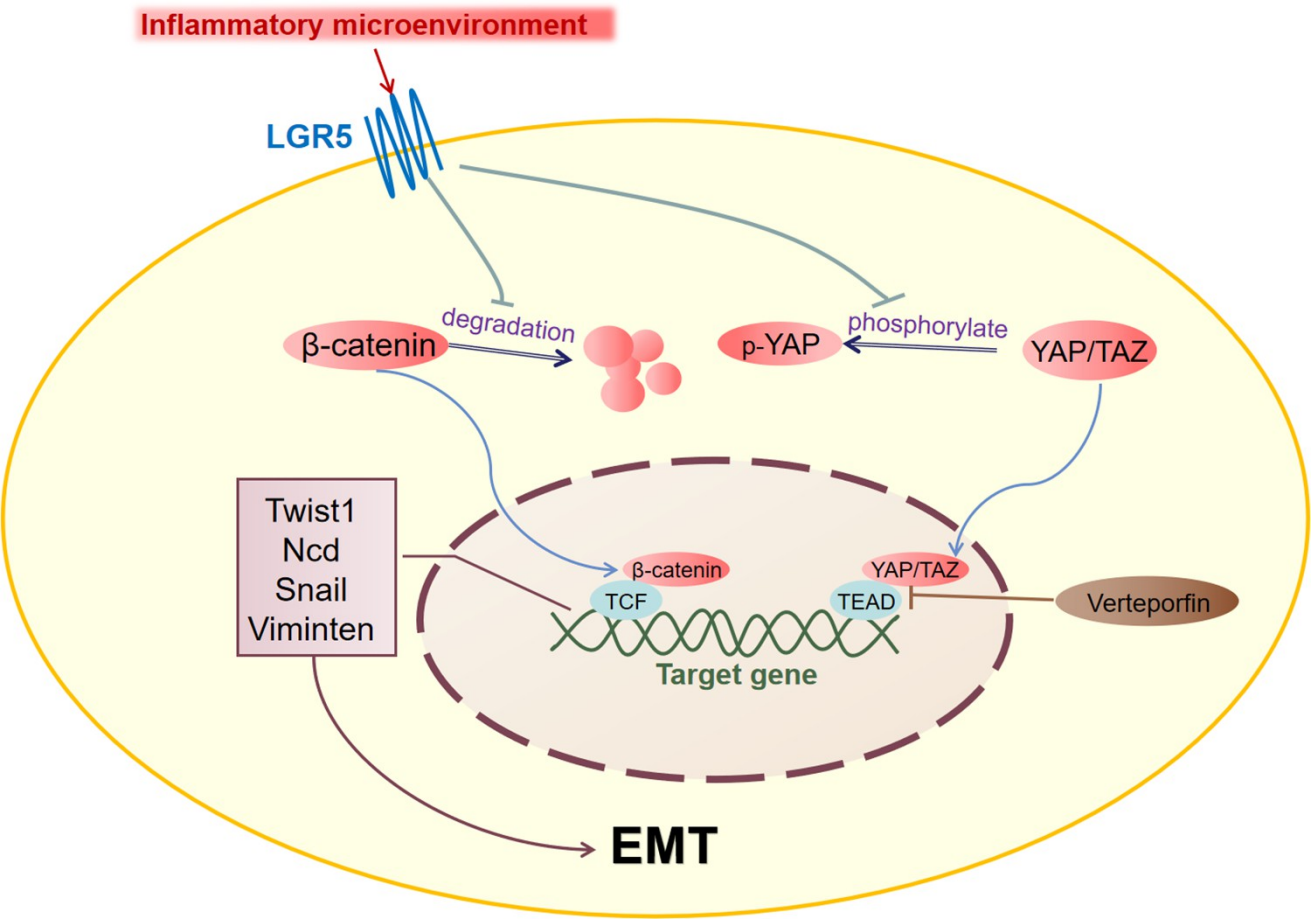
Model for the inflammatory condition results in high expression of LGR5 and tumor cells EMT. LGR5 inhibited β-catenin protein degradation and YAP/TAZ phosphorylation in cytoplasm during hypopharyngeal cancer cells development. Then, β-catenin and YAP/TAZ aggregated in the cytoplasm are introduced into the nucleus and bind to TCF and TEAD family transcription factors, respectively, which together activate transcription of downstream target genes (Twist1, NCD, Snail, Viminten), directly promoting epithelial mesenchymal transformation. Verteprofin(VP) is an effective inhibitor of YAP, which severely interferes with YAP-TEAD interaction.

YAP can be phosphorylated by VP, and phosphorylated YAP binds to proteins and remains in the cytoplasm [39, 40]. Therefore, the destruction of YAP/TAZ nuclear transport by VP can inhibit the EMT process to a certain extent [41], a finding that was also confirmed in this study. In our study, high LGR5 expression could lead to the reduction of p-YAP, and the expression level of EMT-related protein was up-regulated. The use of VP to increase the phosphorylation level of YAP had little effect on LGR5 expression, but the invasion ability of tumor cells was significantly decreased. Therefore, we believe that the inflammatory condition causes high LGR5 expression, which will inhibit the phosphorylation of YAP, resulting in markedly increased YAP-TEAD binding, high expression of EMT-related proteins, and enhanced invasion and metastasis ability of tumor cells. Low expression of the YAP gene by shRNA [42] and interference of the formation of the YAP-TEAD complex by VP [43] have been reported to disrupt cell proliferation and induce apoptosis [44, 45]. In this study, VP was also used to phosphorylate YAP, but many cells died after siRNA inhibition of LGR5 expression; however, few of the cells died in the LGR5 over-expression group (Fig 5A–5f, 5B–5f). In order to determine if LGR5-induced tumor EMT was linked to Hippo system activation, VP was utilized in our investigation as a Hippo signaling pathway inhibitor, utilizing dosages that have been shown to not cause mortality in normally grown FaDu cells. However, we discovered that Fadu cells had undergone apoptosis, nuclear fragmentation, and alterations in cell shape in the experimental group with simultaneous LGR5 siRNA knockdown. Therefore, we assume that the cause of apoptosis in this experimental group is that when the expression of LGR5 is reduced and the phosphorylation of YAP is greatly raised, the growth of Fadu cells is more seriously inhibited, and the cytotoxic outcomes of extensive cell apoptosis are eventually shown. At the same time, we suggest that LGR5 high expression lead to apoptosis resistance and high invasion ability may cause by the phosphorylation inhibition of YAP (Figs 6 and 7).

In conclusion, we have demonstrated for the first time that the inflammatory condition can lead to high LGR5 expression in HSCC, and high LGR5 expression can promote the proliferation and invasion of tumor cells by inhibiting the phosphorylation of YAP. Therefore, LGR5, YAP, and the relationship between LGR5 and YAP phosphorylation can be used as targets for the treatment of HSCC and have potential clinical value in prognosis (Fig 7).

## Supporting information

S1 File Raw data file. (PDF)

S2 File Raw images for western blots. (PDF)

S1 Table Information of qPCR primer sequence. (DOCX)
