# Supplementary material for: LGR5 promotes invasion and migration by regulating YAP activity in hypopharyngeal squamous cell carcinoma cells under inflammatory condition
Source: PLoS One. 2022 Oct 26;17(10):e0275679. doi: 10.1371/journal.pone.0275679 (PMC9604011; doi:10.1371/journal.pone.0275679)
Supplement: S1 File — (PDF) [file pone.0275679.s001.pdf]

Fig1-A

IL-1β		Mean	SD	N
	UT	62.36	34.80	4
	3h	180.75	73.67	4
	6h	664.28	117.66	4
	12h	701.78	115.41	4
	24h	548.59	171.48	4

IL-6		Mean	SD	N
	UT	122.89	20.61	4
	3h	151.57	39.02	4
	6h	711.43	70.58	4
	12h	787.65	176.00	4
	24h	605.85	58.52	4

TNF-α		Mean	SD	N
	UT	478.48	85.61	4
	3h	654.85	93.37	4
	6h	1588.68	315.13	4
	12h	2275.36	686.47	4
	24h	2872.08	293.73	4

Fig1-B

	Mean	SD	N
UT	0.99	0.05	4
24h	1.24	0.04	4

Fig1-D

	UT			FC		
	Mean	SD	N	Mean	SD	N
0h	29.25	4.35	4	28.50	5.07	4
24h	50.00	4.24	4	72.25	5.44	4

Fig1-E

	Mean	SD	N
UT	24.20	7.29	5
FC	55.00	7.07	5

Fig2-B

	Mean	SD	N
UT	0.998	0.132	5
6h	1.098	0.257	5
12h	2.270	0.608	5
24h	2.874	0.731	5

Fig2-D

	Mean	SD	N
UT	0.229	0.050	3
FC 12h	0.834	0.299	3
FC 24h	0.680	0.168	3

Fig3-B

	Con			pReceiver-NC			pReceiver-LGR5		
	Mean	SD	N	Mean	SD	N	Mean	SD	N
Vim	0.863	0.225	3	0.865	0.055	3	1.423	0.046	3
Ncd	0.501	0.209	3	0.461	0.068	3	0.991	0.143	3
Snail	0.688	0.197	3	0.598	0.068	3	1.235	0.221	3

Fig3-D

	FC			FC+siRNA/NC			FC+siRNA/LGR5		
	Mean	SD	N	Mean	SD	N	Mean	SD	N
Ncd	0.975	0.214	3	1.023	0.130	3	0.506	0.004	3
Vim	0.630	0.060	3	0.724	0.241	3	0.293	0.093	3
Snail	0.665	0.109	3	0.742	0.128	3	0.345	0.095	3

Fig4-B

	Mean	SD	N
Con	34.4	5.177	5
pReceiver-NC	30.8	10.402	5
pReceiver-LGR5	68.2	13.405	5

Fig4-D

	Mean	SD	N
Con	33.4	4.506	5
pReceiver-NC	29	7.176	5
pReceiver-LGR5	59.6	6.542	5

Fig4-G

	Mean	SD	N
Con	33	7.517	5
IC	64.8	7.225	5
IC+siRNA/NC	56.2	5.167	5
IC+siRNA/LGR5	28.8	4.147	5

Fig4-H

	Mean	SD	N
IC	56.6	8.620	5
IC+siRNA/NC	52.8	8.167	5
IC+siRNA/LGR5	35	5.612	5

Fig5-E

	Mean	SD	N
siRNA/NC	0.801	0.102	3
VP+siRNA/NC	0.424	0.035	3
siRNA/LGR5	0.511	0.147	3
VP+siRNA/LGR5	0.660	0.052	3

Fig5-F

	Mean	SD	N
siRNA/NC	0.556	0.095	3
VP+siRNA/NC	0.941	0.183	3
siRNA/LGR5	0.981	0.086	3
VP+siRNA/LGR5	0.805	0.098	3

Fig5-G

	Mean	SD	N
Con	0.462	0.062	3
VP+LGR5+	0.800	0.069	3
LGR5+	0.744	0.092	3

Fig5-H

	Mean	SD	N
Con	0.988	0.193	3
VP+LGR5+	1.119	0.129	3
LGR5+	0.571	0.054	3

Fig6-B

	LGR5+			VP+LGR5+		
	Mean	SD	N	Mean	SD	N
0h	40.333	3.055	3	41	2	3
24h	70.667	3.786	3	47.667	5.508	3

Fig6-D

	Mean	SD	N
LGR5+	131.00	14.53	3
VP+LGR5+	40.33	4.16	3
