# Supplementary material for: LGR5 promotes invasion and migration by regulating YAP activity in hypopharyngeal squamous cell carcinoma cells under inflammatory condition
Source: PLoS One. 2022 Oct 26;17(10):e0275679. doi: 10.1371/journal.pone.0275679 (PMC9604011; doi:10.1371/journal.pone.0275679)
Supplement: S2 File — (PDF) [file pone.0275679.s002.pdf]

Fig-2C

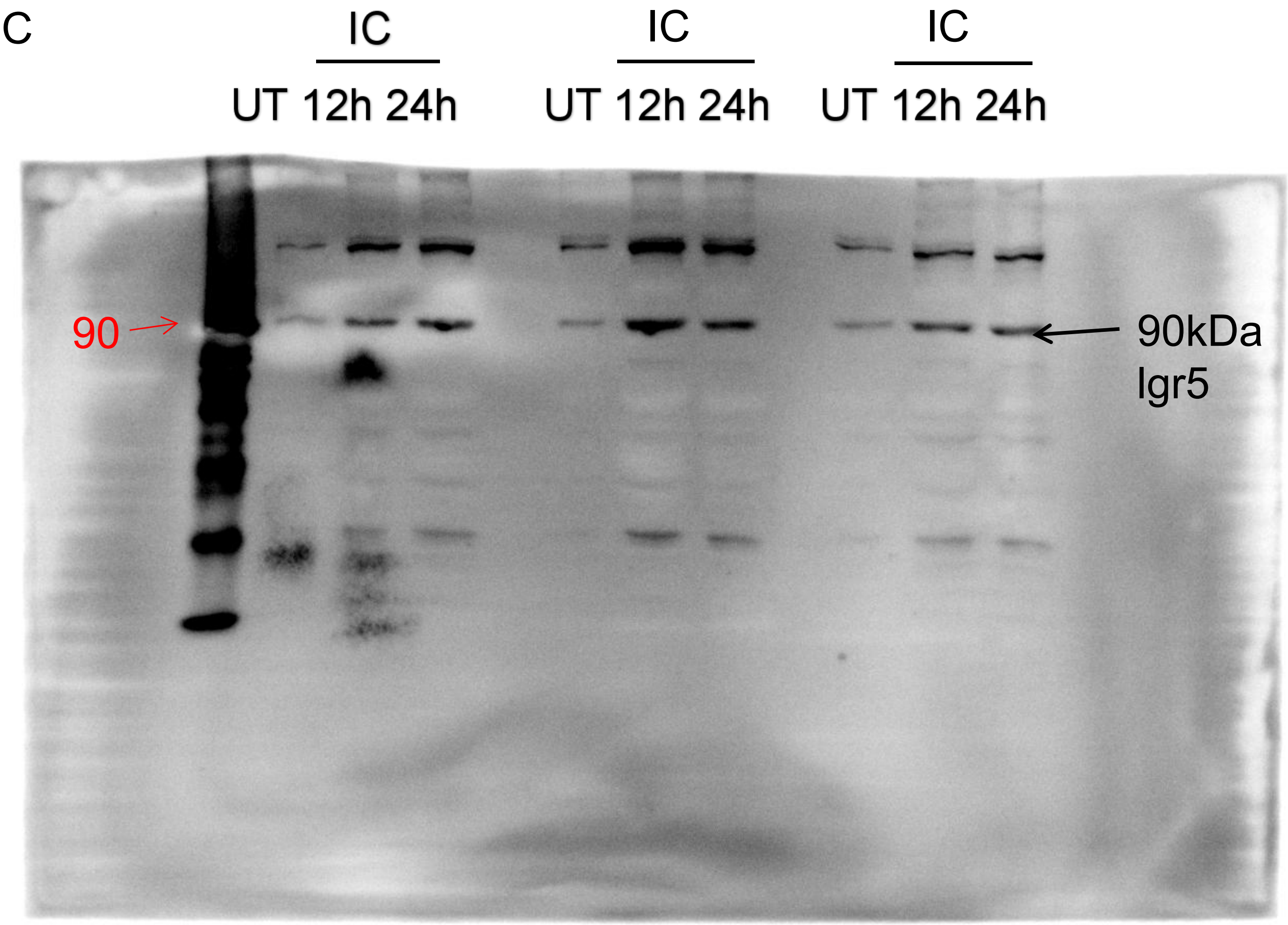

Fig-3A

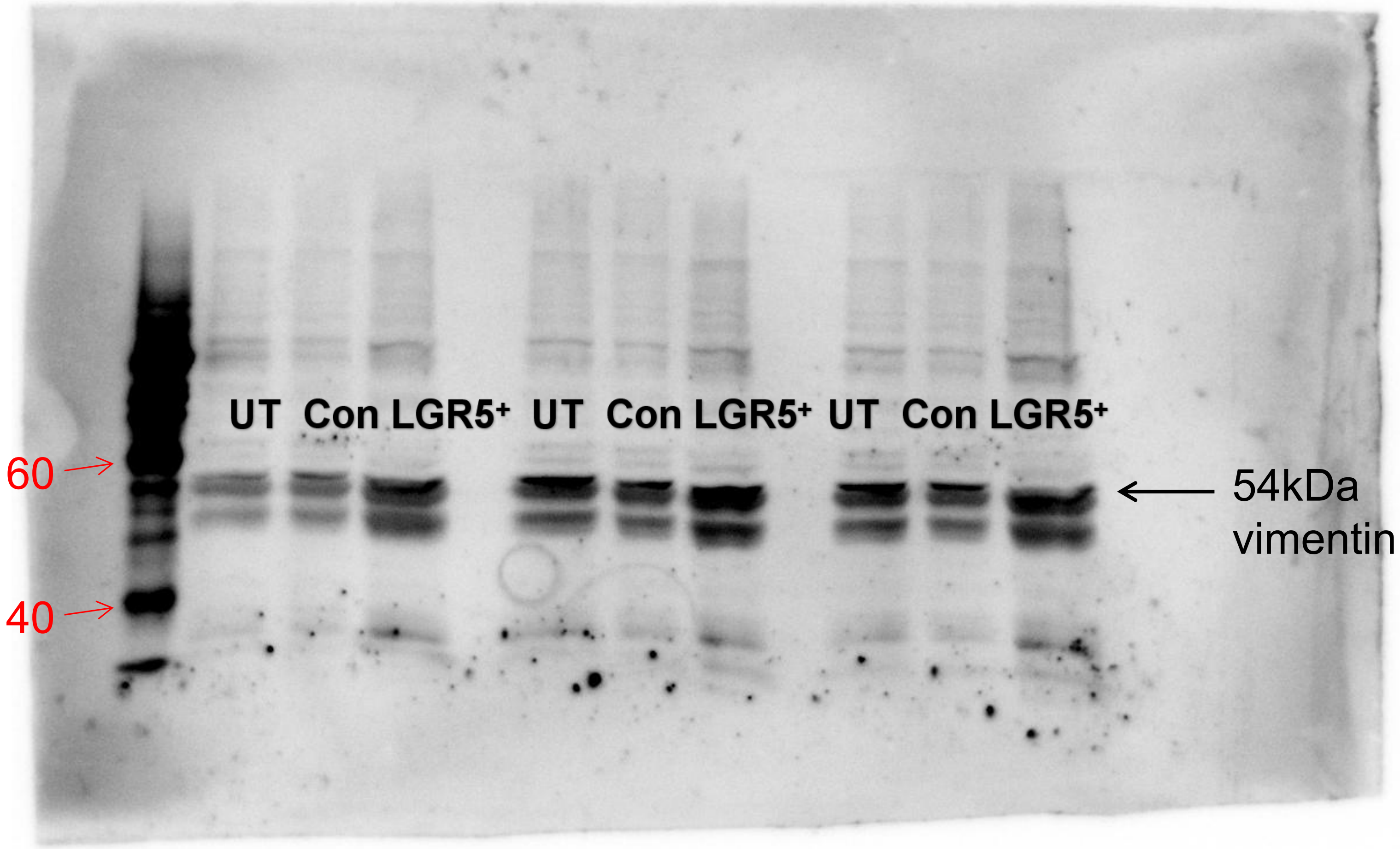

Fig-3A

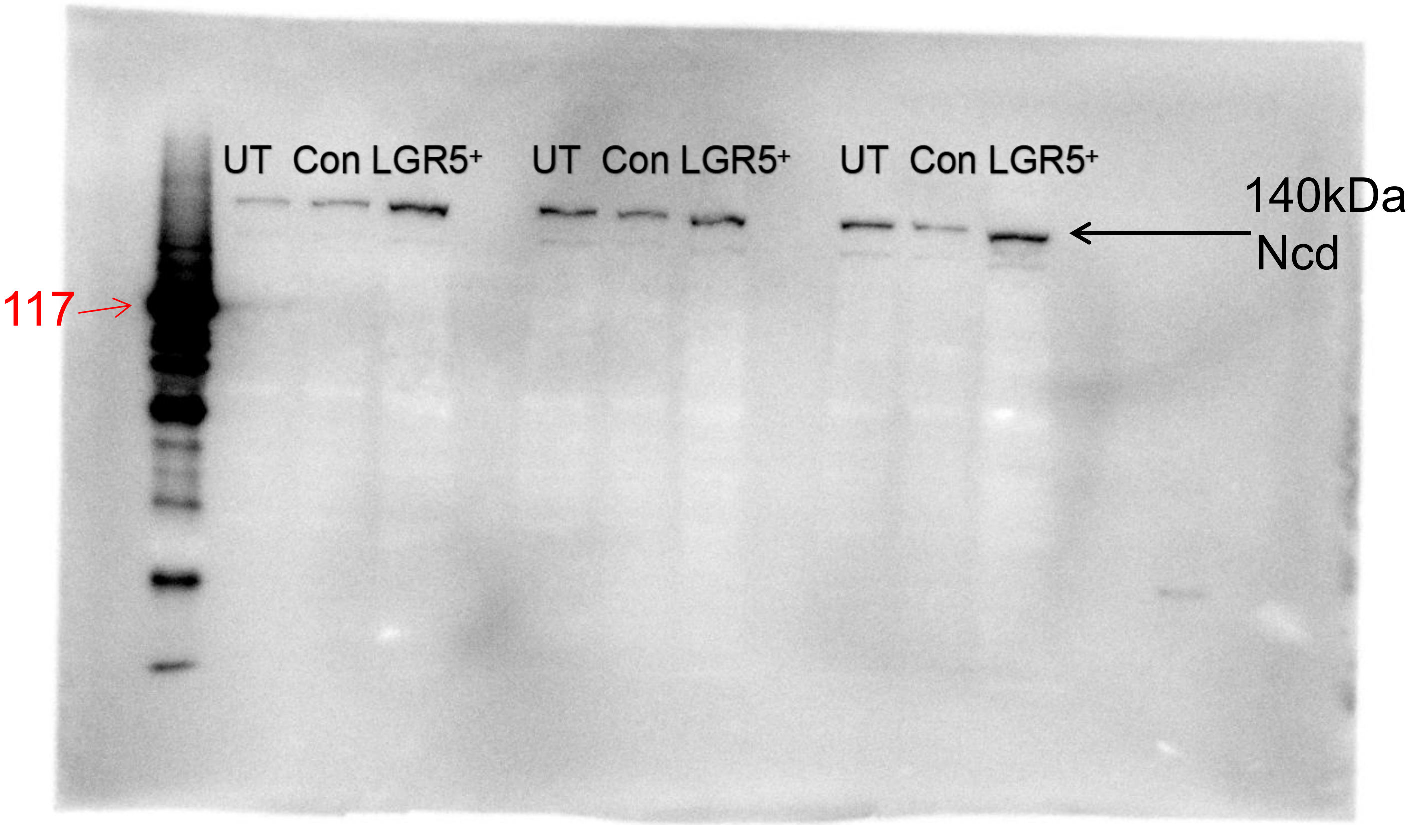

Fig-3A

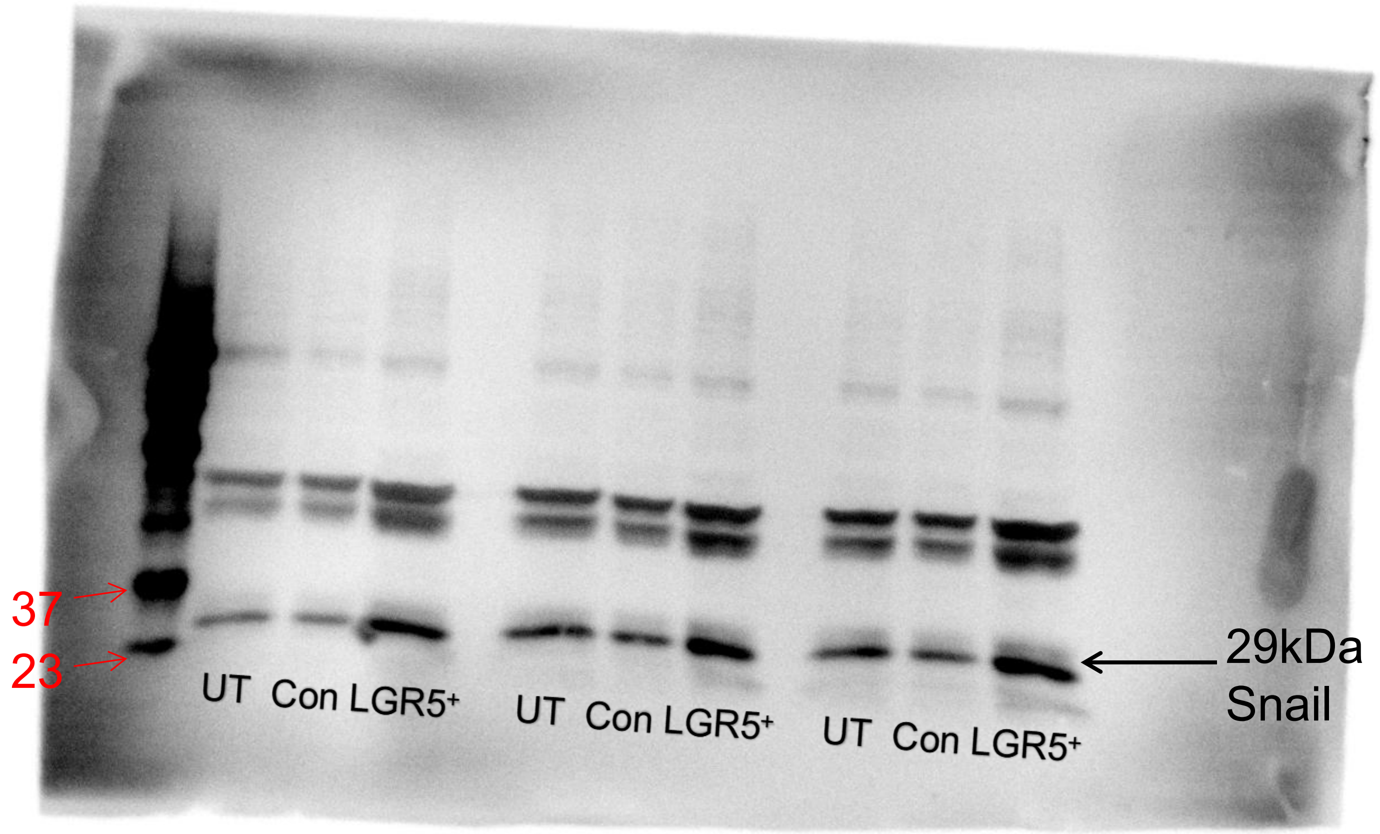

Fig-3C

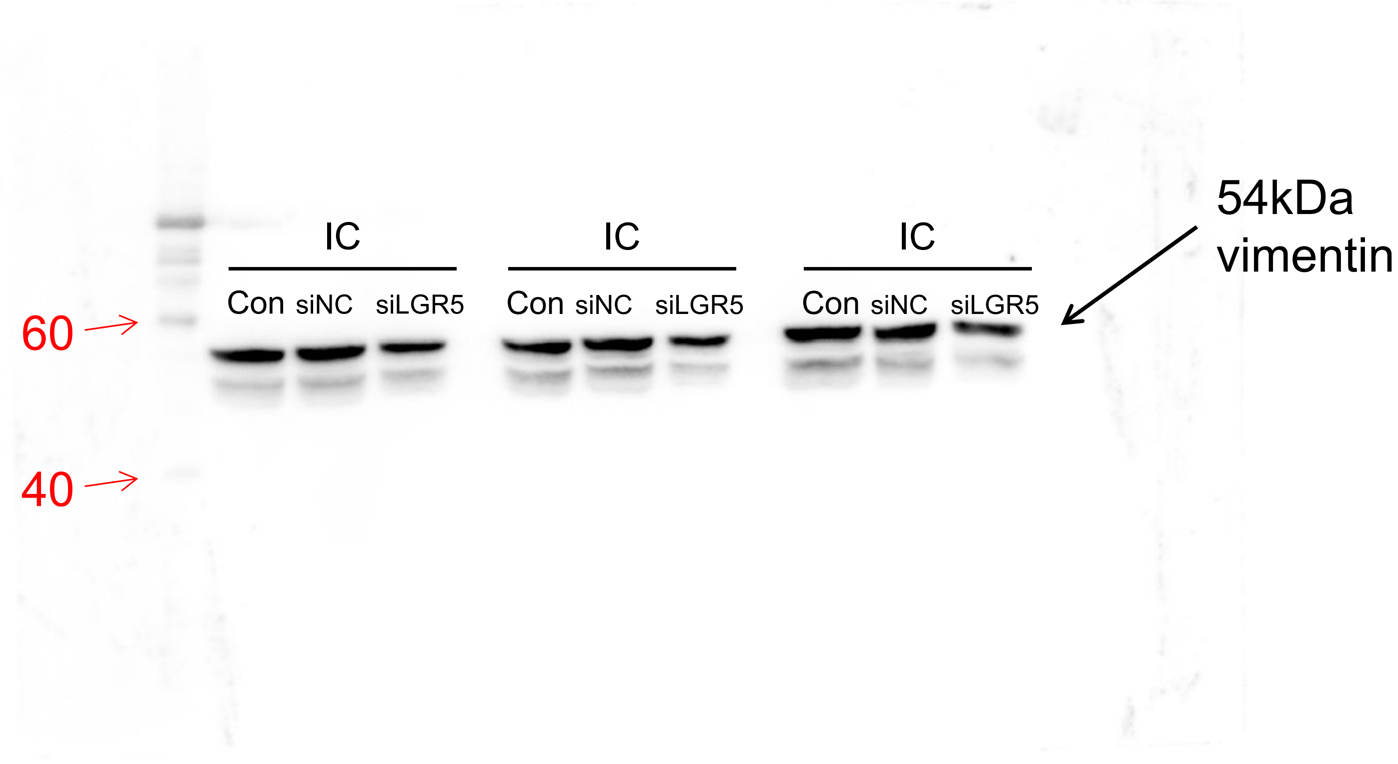

IC
IC
IC
Con siNC siLGR5
Con siNC siLGR5
Con siNC siLGR5
60
40
54kDa
vimentin

Fig-3C

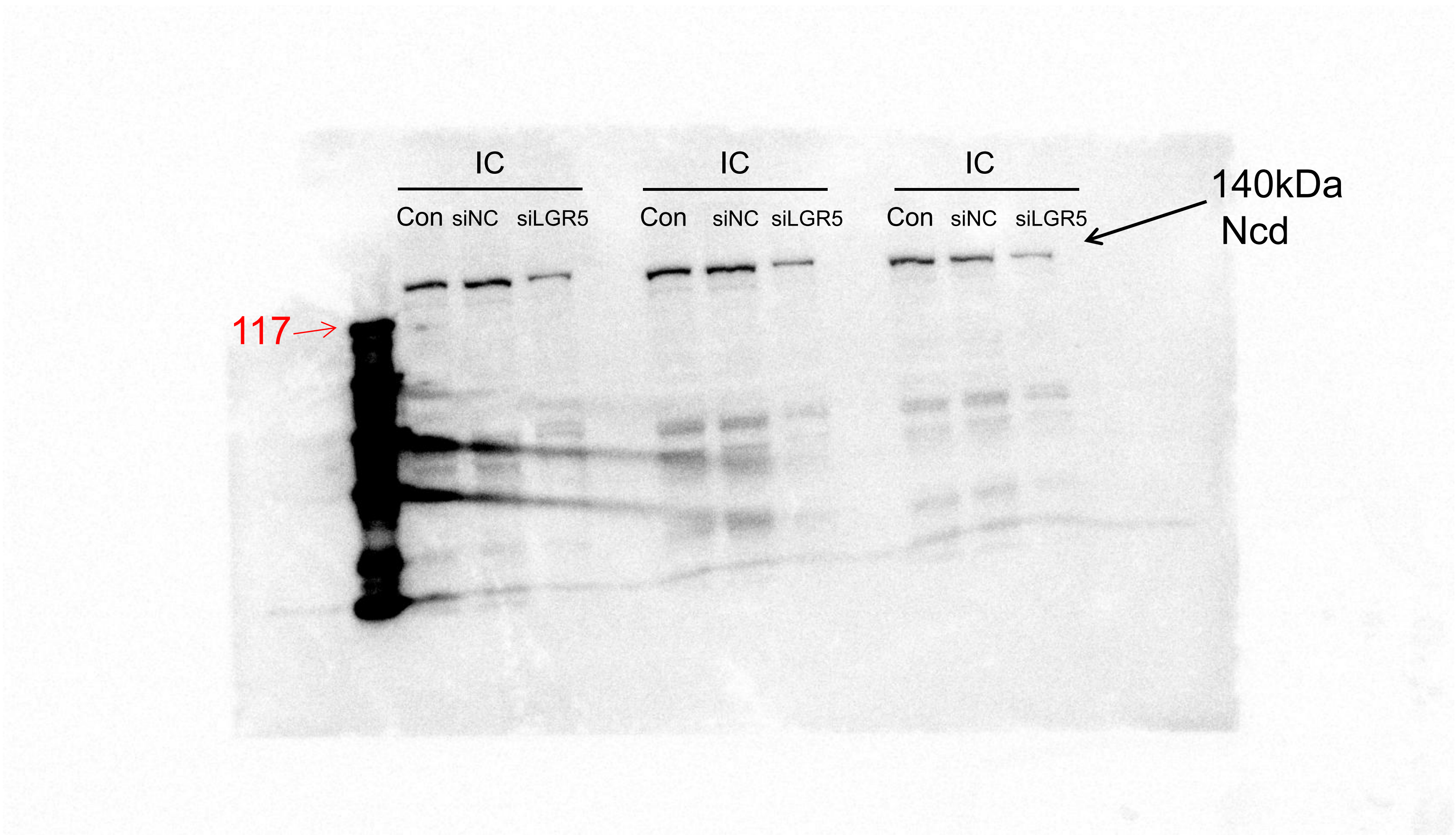
IC
IC
IC
Con siNC siLGR5
Con siNC siLGR5
Con siNC siLGR5
140kDa
Ncd
117

Fig-3C

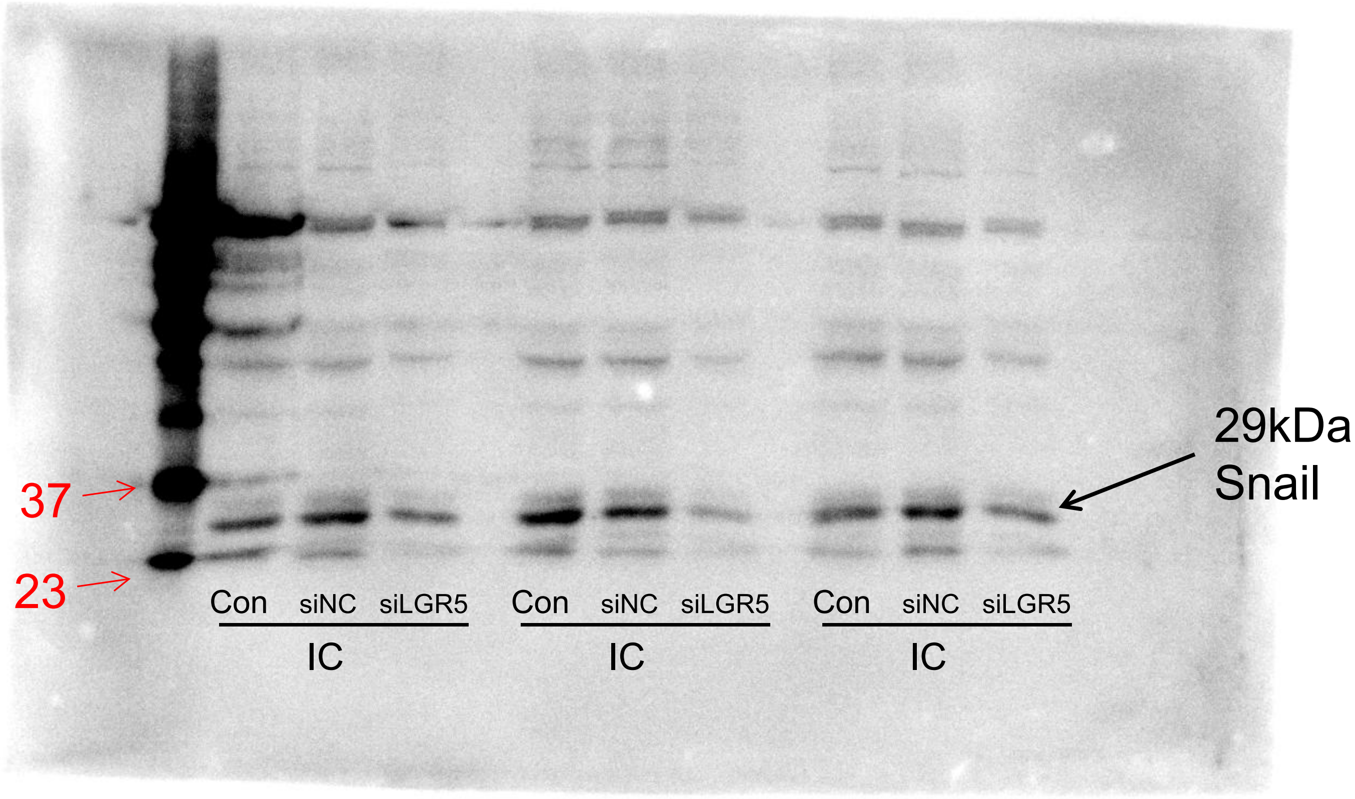
37
23
Con
siNC
siLGR5
Con
siNC
siLGR5
Con
siNC
siLGR5
IC
IC
IC
29kDa
Snail

Fig-5C

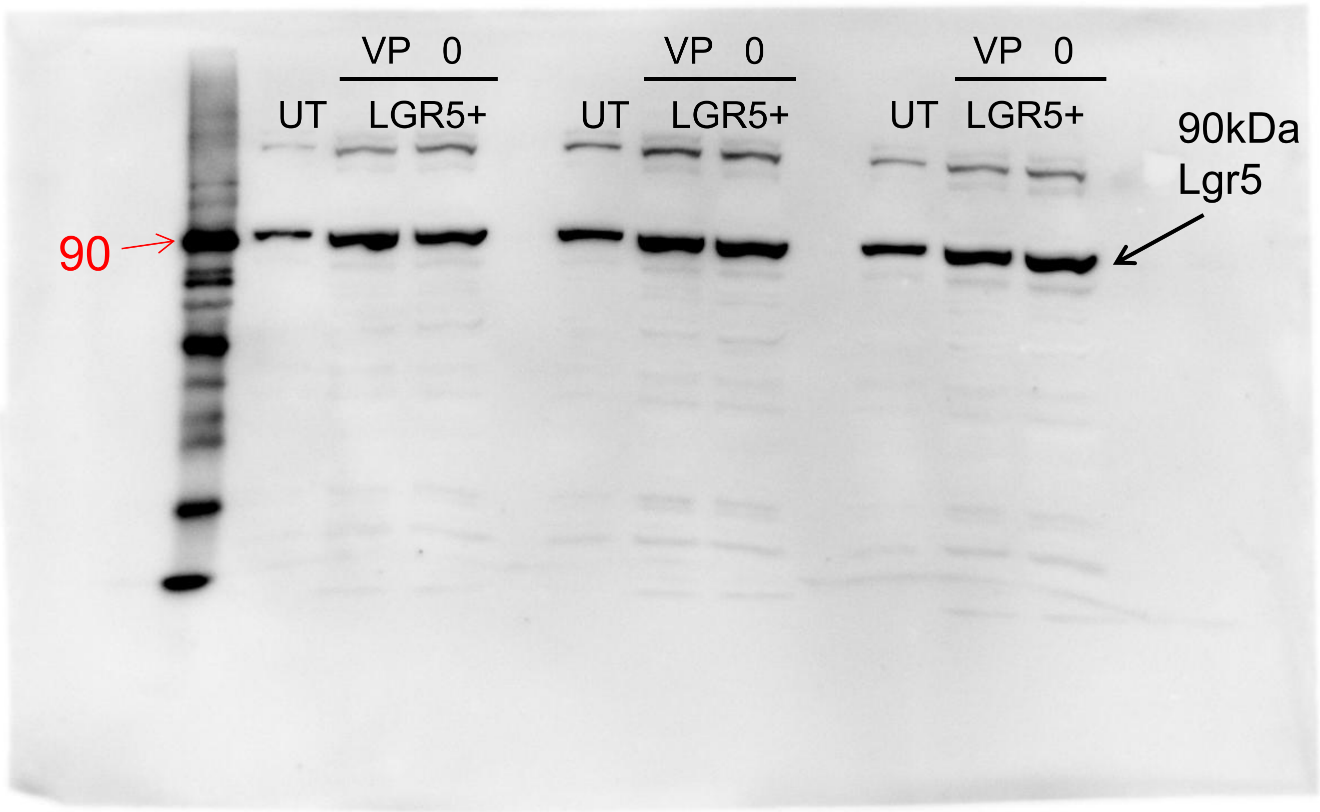

Fig-5C

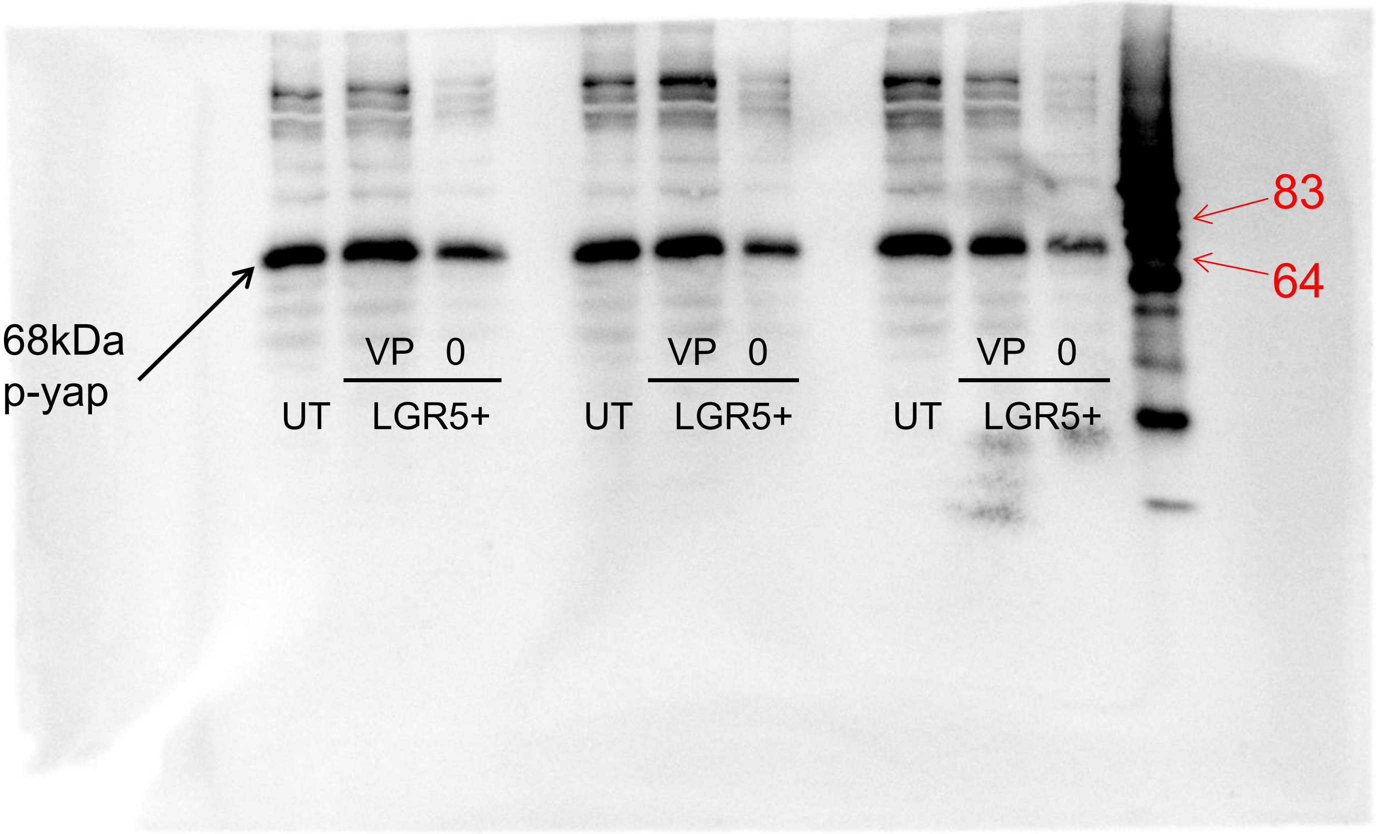

Fig-5C

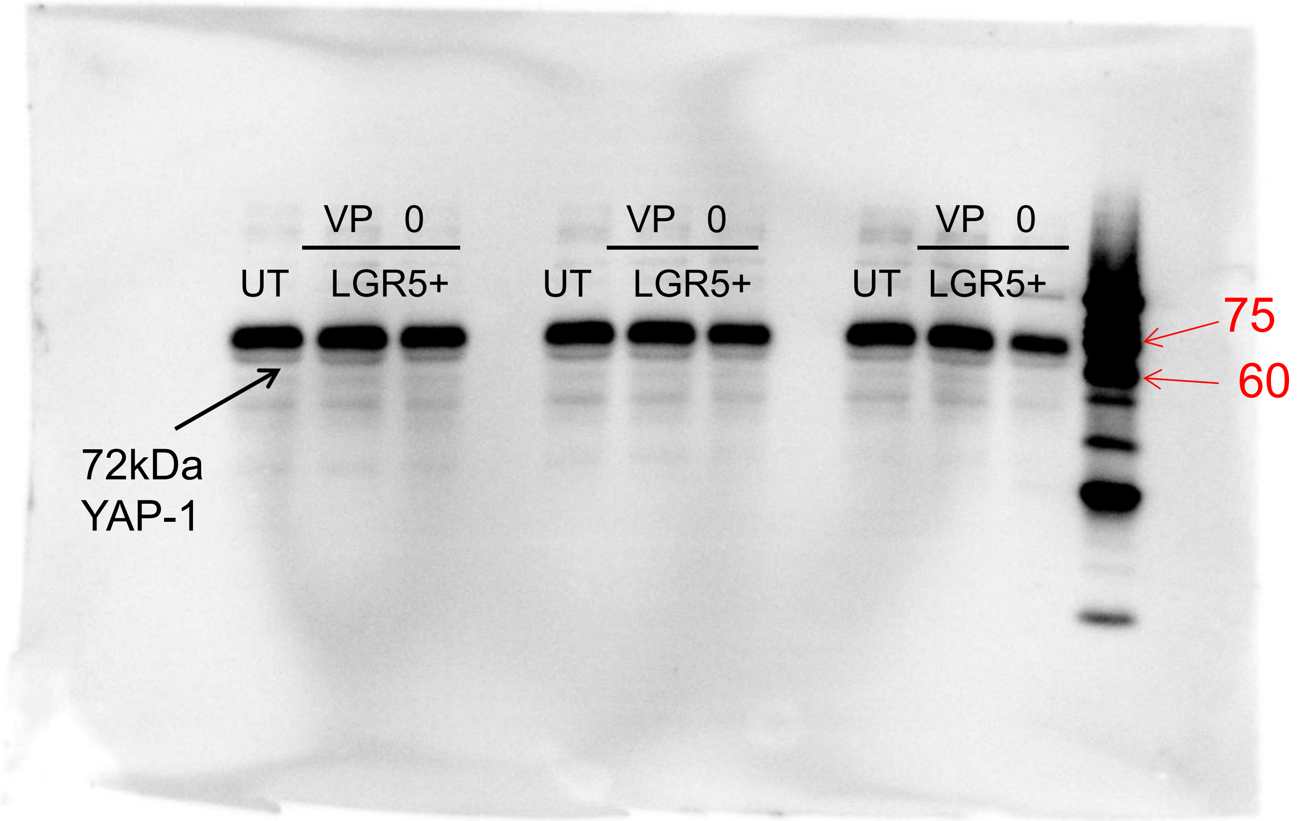

Fig-5D

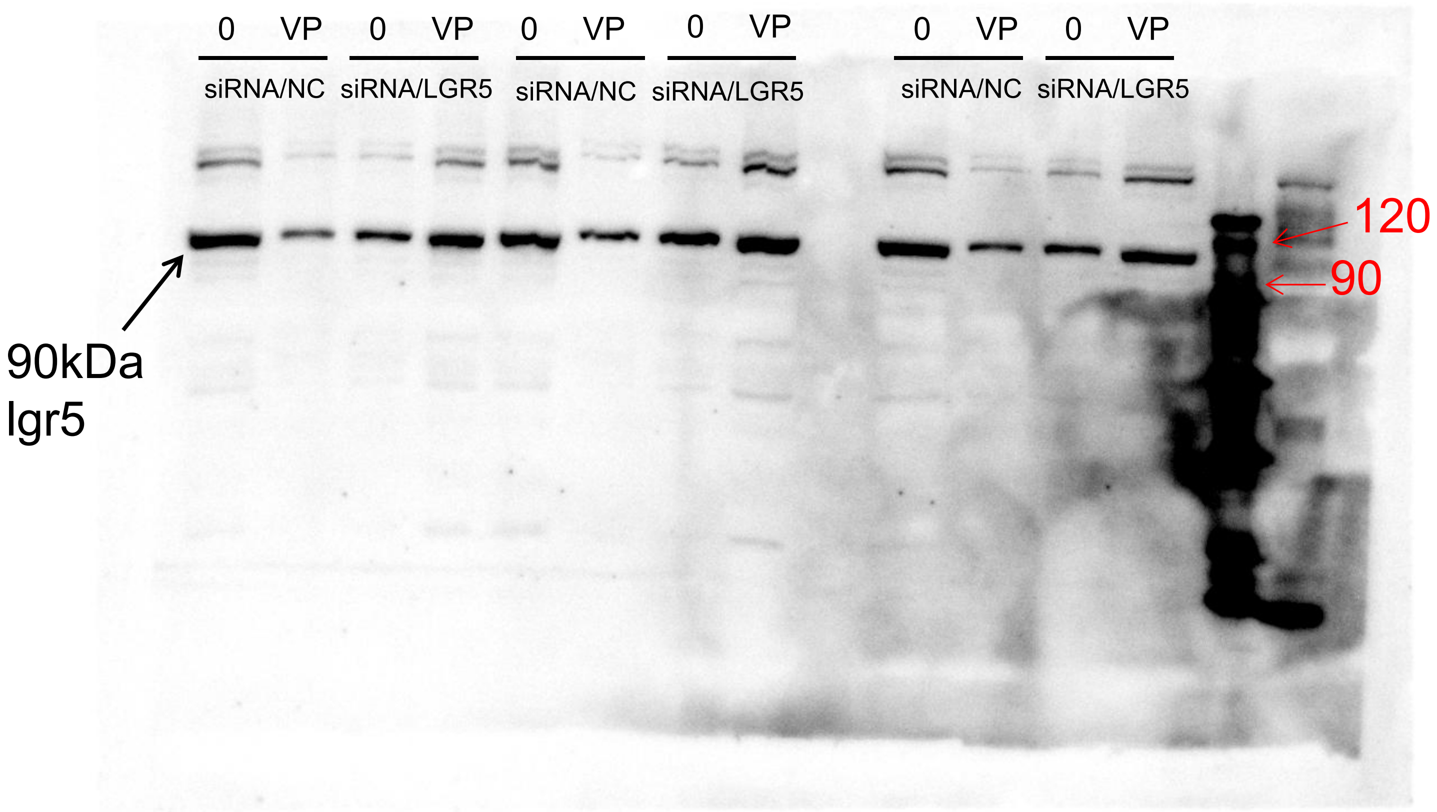
0
VP
0
VP
0
VP
0
VP
0
VP
0
VP
siRNA/NC
siRNA/LGR5
siRNA/NC
siRNA/LGR5
siRNA/NC
siRNA/LGR5
120
90
90kDa
lgr5

Fig-5D

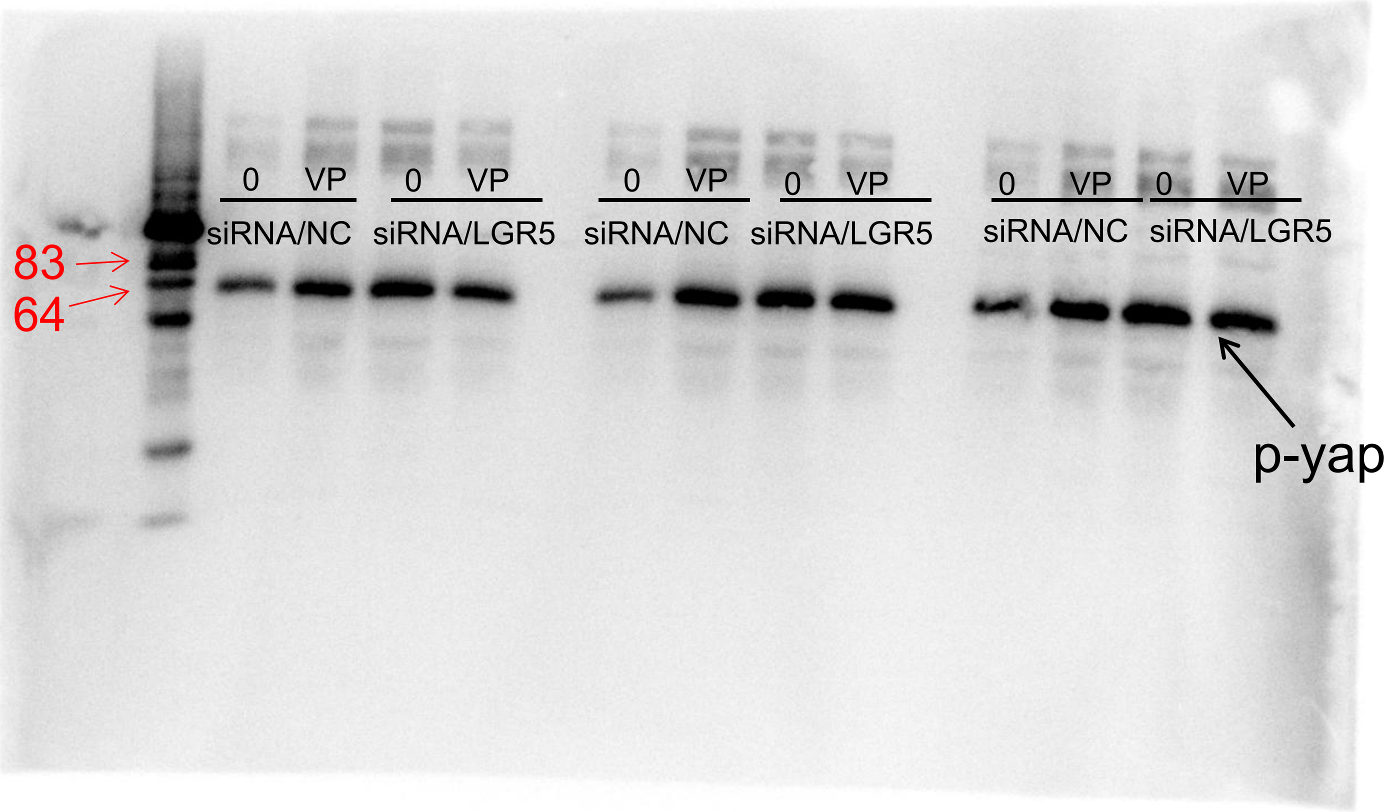
0
VP
0
VP
0
VP
0
VP
0
VP
0
VP
siRNA/NC
siRNA/LGR5
siRNA/NC
siRNA/LGR5
siRNA/NC
siRNA/LGR5
83
64
p-yap

Fig-5D

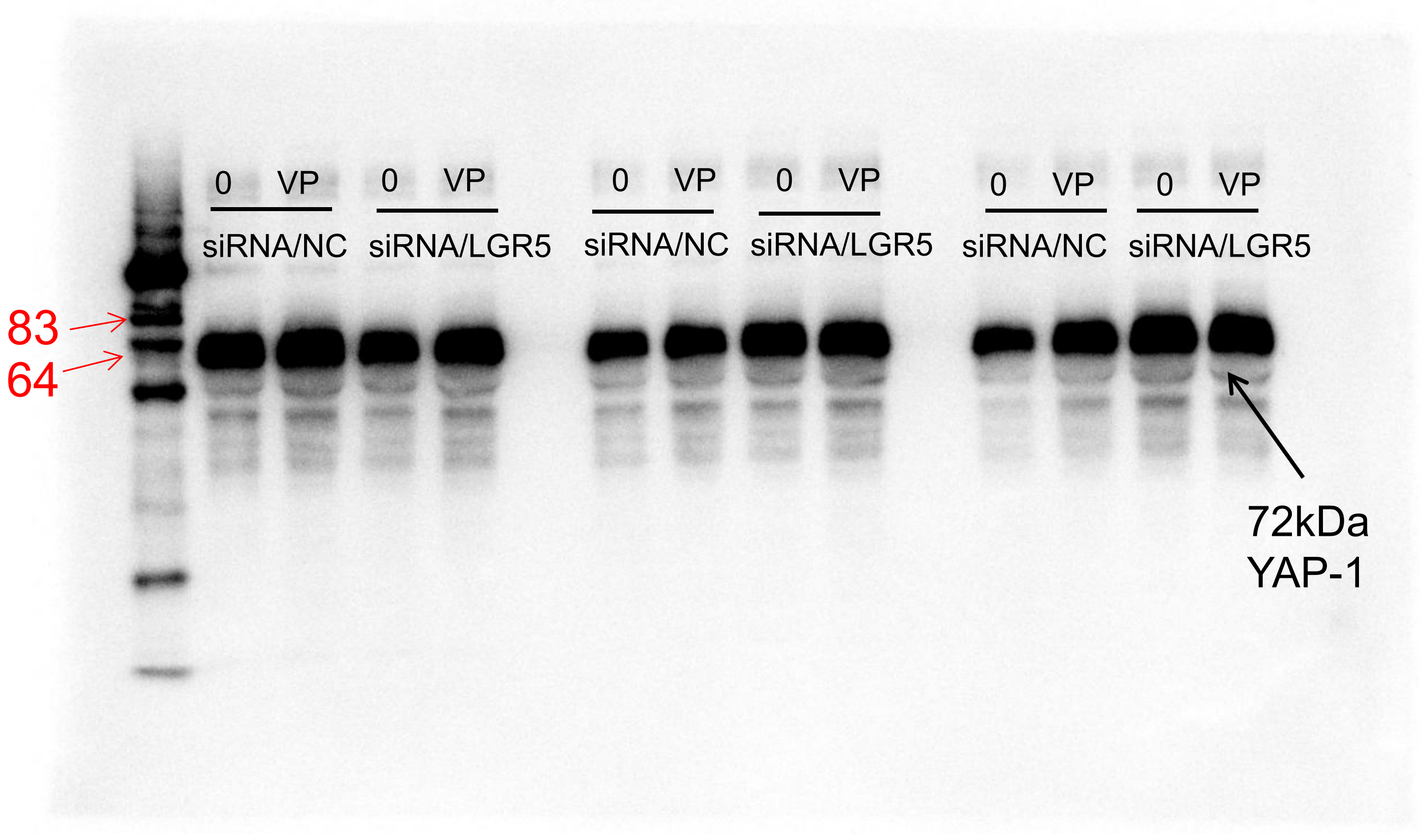
0
VP
0
VP
0
VP
0
VP
0
VP
0
VP
siRNA/NC
siRNA/LGR5
siRNA/NC
siRNA/LGR5
siRNA/NC
siRNA/LGR5
83
64
72kDa
YAP-1
