# Supplementary material for: LGR5 promotes invasion and migration by regulating YAP activity in hypopharyngeal squamous cell carcinoma cells under inflammatory condition
Source: PLoS One. 2022 Oct 26;17(10):e0275679. doi: 10.1371/journal.pone.0275679 (PMC9604011; doi:10.1371/journal.pone.0275679)
Supplement: S1 Table — (DOCX) [file pone.0275679.s003.docx]

| Gene name | Forward Primer | Reverse Primer |
| --- | --- | --- |
| LGR5 | 5’-CCTGCTTGACTTTGAGGAAGACC-3’ | 5’-CCAGCCATCAAGCAGGTGTTCA-3’ |
| Snail1 | 5'-TTTCTGGTTCTGTGTCCTCTG-3' | 5'-TGTCAGCCTTTGTCCTGTAGC-3' |
| Twist1 | 5'-AGTCCGCAGTCATACGAGGAG-3' | 5'-GACCTAGTAGAGGAAGTCGATG-3' |
| Vimentin | 5’-AGTCCACTGAGTACCGGAGAC-3’ | 5’-CATTTCACGCATCTGGCGTTC-3’ |
| Yap | 5’–GCTACAGTGTCCCTCGAACC–3’ | 5’-CCGGTGCATGTGTCTCCTTA-3’ |
| β-actin | 5'-TGGCACCCAGCACAATGAA-3' | 5'- CTAAGTCATAGTCCGCCTAG-3' |
